# Reproductive Incompatibility Involving Senegalese *Aedes aegypti* (L) Is Associated with Chromosome Rearrangements

**DOI:** 10.1371/journal.pntd.0004626

**Published:** 2016-04-22

**Authors:** Laura B. Dickson, Maria V. Sharakhova, Vladimir A. Timoshevskiy, Karen L. Fleming, Alex Caspary, Massamba Sylla, William C. Black

**Affiliations:** 1 Department of Microbiology, Immunology and Pathology, Colorado State University, Fort Collins, Colorado, United States of America; 2 Department of Entomology, Fralin Life Science Institute, Virginia Tech, Blacksburg, Virginia, United States of America; Mahidol University, THAILAND

## Abstract

*Aedes aegypti*, the primary vector of dengue, yellow fever and Zika flaviviruses, consists of at least two subspecies. *Aedes aegypti* (*Aaa*) is light in color, has pale scales on the first abdominal tergite, oviposits in artificial containers, and preferentially feeds on humans. *Aedes aegypti formosus* (*Aaf*), has a dark cuticle, is restricted to sub-Saharan Africa, has no pale scales on the first abdominal tergite and frequently oviposits in natural containers. Scale patterns correlate with cuticle color in East Africa but not in Senegal, West Africa where black cuticle mosquitoes display a continuum of scaling patterns and breed domestically indoors. An earlier laboratory study did not indicate any pre- or postzygotic barriers to gene flow between *Aaa* and *Aaf* in East Africa. However, similar attempts to construct F_1_ intercross families between *Aaa* laboratory strains and Senegal *Ae*. *aegypti* (*SenAae*) failed due to poor F_1_ oviposition and low F_2_ egg-to-adult survival. Insemination and assortative mating experiments failed to identify prezygotic mating barriers. Backcrosses were performed to test for postzygotic isolation patterns consistent with Haldane’s rule modified for species, like *Aedes*, that have an autosomal sex determining locus (SDL). Egg-pupal survival was predicted to be low in females mated to hybrid F_1_ males but average when a male mates with a hybrid F_1_ female. Survival was in fact significantly reduced when females mated to hybrid males but egg-pupal survival was significantly increased when males were mated to hybrid F_1_ females. These observations are therefore inconclusive with regards to Haldane’s rule. Basic cytogenetic analyses and Fluorescent In Situ Hybridization (FISH) experiments were performed to compare *SenAae* strains with the IB12 strain of *Aaa* that was used for genome sequencing and physical mapping. Some *SenAae* strains had longer chromosomes than IB12 and significantly different centromeric indices on chromosomes 1 and 3. DAPI staining was used to identify AT-rich regions, chromomycin A3 following pretreatment with barium hydroxide stained for GC-rich regions and stained the ribosomal RNA locus and YOYO-1 was used to test for differential staining. Chromosome patterns in *SenAae* strains revealed by these three stains differed from those in IB12. For FISH, 40 BAC clones previously physically mapped on *Aaa* chromosomes were used to test for chromosome rearrangements in *SenAae* relative to IB12. Differences in the order of markers identified two chromosomal rearrangements between IB12 and *SenAae* strains. The first rearrangement involves two overlapping pericentric (containing the centromere) inversions in chromosome 3 or an insertion of a large fragment into the 3q arm. The second rearrangement is close to the centromere on the p arm of chromosome 2. Linkage analysis of the SDL and the *white-eye* locus identified a likely chromosomal rearrangement on chromosome 1. The reproductive incompatibility observed within *SenAae* and between *SenAae* and *Aaa* may be generally associated with chromosome rearrangements on all three chromosomes and specifically caused by pericentric inversions on chromosomes 2 and 3.

## Author Summary

*Aedes aegypti* is one of the best studied mosquito species and it is the principal vector of dengue, Zika, and yellow fever flaviviruses and the Chikungunya alphavirus. *Aedes aegypti* occurs throughout all tropical and subtropical regions of the world, and previous population genetic studies have shown that the highest genetic diversity occurs in Africa. *Aedes aegypti* from Senegal, West Africa (*SenAae*) have a low oviposition rate; those that do oviposit have a low fecundity and poor egg-to-adult survival. Furthermore rearrangements were detected on all three chromosomes in *SenAae*. These observations are consistent with the presence of at least two cryptic subspecies of *Ae*. *aegypti* in Senegal arising from reproductive isolation due to chromosome rearrangements. Genetic control strategies are being considered for the suppression of *Ae*. *aegypti* populations worldwide. Barriers to gene flow in African *Ae*. *aegypti* populations could compromise these future control efforts.

## Introduction

The mosquito, *Aedes aegypti* (L), is the principal vector of dengue (DENV1-4) [1, 2], Yellow Fever (YF) [3, 4] and Zika [5, 6] flaviviruses in tropical and subtropical regions world-wide. The ecology and population biology of the species have been studied since the mid-1950s [7–10]. At that time, *Ae*. *aegypti* in East Africa was known to have a high frequency of pale cuticle forms that preferred peridomestic sites and readily fed on humans and dark cuticular forms that predominated in the nearby bush and more readily fed upon wild animals [9, 10]. This correlation between body color and behavior prompted Mattingly to revisit the biology and taxonomy of *Ae*. *aegypti* [11] wherein he defined the type form, *Ae*. *aegypti* (*Aaa*), as having a global tropical and subtropical distribution, a light-tan cuticle, pale scales on the first abdominal tergite, a feeding preference for humans and which laid its eggs in artificial containers (e.g. tires, discarded jars). The dark form was described as a new subspecies, *Ae*. *aegypti formosus* (Walker) (*Aaf*) [11] that was restricted to sub-Saharan Africa, laid its eggs in natural containers (e.g. tree holes), had adults without pale scales on the first abdominal tergite, and only rarely fed upon humans [8]. Later, mark-release-recapture studies in Kenya [12] demonstrated that immature mosquitoes collected from sylvan, peridomestic or domestic breeding containers showed an overwhelming preference for their respective habitats as adults. A 1979 study of hybridization and mating behavior between *Aaa* and *Aaf* in East Africa found no evidence of hybrid breakdown or of assortative mating between the two subspecies and concluded that they are part of a single, albeit highly polytypic species[13]. A recent study [14] in East Africa nicely illustrates the differences in color between *Aaa* and *Aaf* and reaffirms the correlation between feeding preference for humans, lighter cuticle and the quantity of pale scales on the first abdominal tergite. Furthermore preference for humans in *Aaa* was tightly linked to increases in the expression and sensitivity of an odorant receptor.

Population genetic studies in the early 1970’s and continuing to the present [15–23] have consistently indicated that *Aaa* and *Aaf* subspecies are genetically distinct [21], that both originated in Africa but that *Aaa* is the form that has spread globally whereas *Aaf* has remained an African endemic taxon [22]. In early population genetic studies it was assumed that *Ae*. *aegypti* from West Africa were also *Aaf* until McClelland performed a comprehensive study of the differences in scaling pattern in 69 different worldwide collections [18]. He found collections of almost pure *Aaf* in Pensacola, Key West and Miami, Florida. Conversely, collections from Kenya, Nigeria, Tanzania, Senegal, Ghana, Burkina Faso, Sri Lanka, Calcutta, Jamaica, and the Miami Airport contained diverse mixtures of *Aaf* and *Aaa* mosquitoes. He concluded that distinctions between the subspecies based on body color, scaling and behavior were not definitive, even within Africa, the only region in the world where both forms are found. While the presence or absence of scales correlates with genetic differences in East Africa [21] this is not the case in West Africa [19, 24–27] where cuticle color is predominantly black but mosquitoes exhibit a continuum of scaling patterns. In addition, *Aaf* were found breeding domestically indoors in Nigeria [28] and Gabon [29]. Collections from West Africa that varied in scaling patterns were sampled in both the dry and rainy seasons from different vegetation zones and from domestic versus sylvan habitats. They were compared using allozyme markers [24], microsatellites [16, 25, 26], nuclear SNP loci [15, 17, 27] and mitochondrial DNA [19, 25]. Regardless of collection site or marker type, there was little variance in marker frequencies between mosquitoes with or without scales. Instead most variation was associated with geographic distance, vegetative zones, ecological habitats and season [15, 16, 24, 25, 27]. Collectively these studies suggest a more complex and unresolved genetic structure in West African *Ae*. *aegypti*.

With the goal of performing Quantitative Trait Locus (QTL) mapping of gene regions associated with DENV2 susceptibility, *Aaa* P_1_ laboratory strains that are known to be highly susceptible to DENV2 infection were crossed with Senegal *Ae*. *aegypti (SenAae)* that are refractory to DENV2 infection [30] to breed F_1_ intercross families. During this process reproductive incompatibilities were observed between *Aaa* collected outside Africa and *SenAae*. Furthermore reproductive incompatibilities were observed within and among *SenAae* collections. These observations differ markedly from the 1979 study of hybridization and mating behavior between *Aaa* and *Aaf* in East Africa which found no evidence of hybrid breakdown or of assortative mating [11]. These leads to the hypothesis that, unlike *Aaa* and *Aaf*, *Aaa* and *SenAae* are reproductively incompatible. Herein possible biological and genetic causes for the observed incompatibilities within *SenAae* and between *Aaa* and *SenAae* are explored.

Throughout this study the lab strains of *Ae*. *aegypti* (D2S3 [31], ROCK [32], IB12 [33]) are designated as *Aaa* because they fit Mattingly’s original description [11]. *SenAae* that had any scales on the first abdominal tergite are designated as “G” (following McClelland’s nomenclature [18]) rather than *Aaa* because while they have scales they also have a black cuticle which does not fit the original *Aaa* description [11]. To date light or tan cuticle *Ae*. *aegypti* have not been detected in Senegal (and these differences are very obvious (see Fig 1d in [14]). *SenAae* without scales on the first abdominal tergite are designated as *“*F*”* [18] rather than *Aaf* because earlier studies showed no reproductive isolation between *Aaa* and *Aaf* in East Africa [13, 21].

These studies, by necessity collectively involved eight different field-collected *SenAae* strains. Use of different strains was necessary because of the continuous loss of strains due to poor initial oviposition rates, high larval mortality and low F_1_ adult fecundity. This prohibited the consistent use of the same *SenAae* strains in all experiments.

## Methods

### Mosquito collections

**Fig 1:**
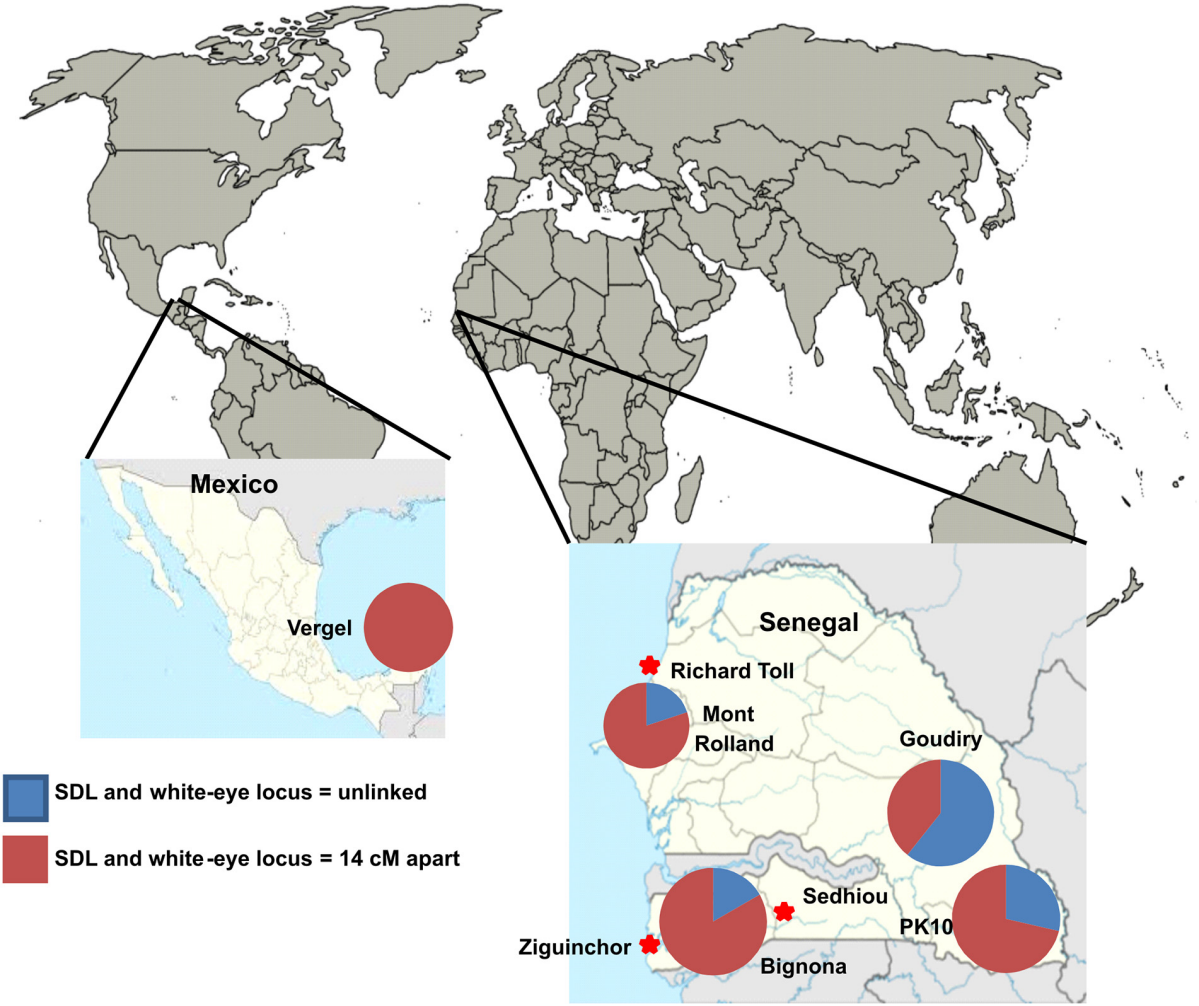
Map of all collection sites used in the present study. Longitude and latitudes of each site are listed in Table 1. Pie charts indicate the proportions of families with SDL and white-eye linked (red) or unlinked (blue).

The D2S3 lab strain of *Ae*. *aegypti* [31] originated from crosses between Puerto Rico and Ibo, Nigeria parents followed by selection for mosquitoes with high DENV2 disseminated infection rates and ROCK [32] which originated from Cuba [34]. *SenAae* populations were collected as larvae near domestic sites in urban and village environments, as well as from sylvatic habitats throughout Senegal [27] (Table 1 and Fig 1). A complete description of mosquito species found in PK10 during the dry and wet seasons has been published [35].

**Table 1:** Location, month and year that the eight Senegal collections and one Mexican collection were made. Scaling ranged from mixed in collections containing mosquitoes with a wide range of scaling patterns on the first abdominal tergite to no scales in PK10 to all scaled in Mexico. In PK10 forest, the abundance of mosquitoes with and without scales differed seasonally.

Country	Collection	Month	Year	Latitude	Longitude	Scaling
Senegal						
	Sedhiou	February	2010	12°42'16.57"N	15°33'22.42"W	Mixed
	Bignona	February	2010	12°48'18.42"N	16°14'4.32"W	Mixed
	Ziguinchor	February	2010	12°34'47.78"N	16°17'2.22"W	Mixed
	PK10 forest	February	2010	12°36'43.00"N	12°14'46.80"W	Mostly *Aaa*
	Mont Rolland	July	2010	14°55'10.96"N	16°59'28.75"W	Mixed
	Richard Toll	July	2010	16°27'40.55"N	15°41'15.51"W	Mixed
	Kedougou			12°33'23.22"N	12°10'48.10"W	
	PK10 forest	September	2011	12°36'43.00"N	12°14'46.80"W	No scales
	Goudiry	September	2011	14°10'60.00"N	12°43'0.00"W	Mixed
Mexico						
	Merida (Vergel)	September	2007	20°57'16.82"N	89°35'20.24"W	Mostly *Aaf*

In each collection, larvae were removed mostly from artificial containers (e.g. tires, water storage containers, discarded trash), reared to adults, blood fed on the senior author’s arm and collected eggs were returned to Fort Collins where they were established as laboratory colonies. The PK10 colonies were established from larvae taken from treeholes at the forest-savannah margin [35]. In each collection, these were raised to adults, transferred to half liter cages, anesthetized with Triethylamine (FlyNap Carolina Biological Supply Company, Burlington, NC) and classified according to the presence/absence of scales on the first abdominal tergite [18, 27, 35]. At least 50 adults of each sex were individually identified and stored in Purell Advanced Hand Sanitizer for eventual extraction of DNA [36]. All colonized adults were maintained at 28°C, 70–80% relative humidity and for a 12:12 hour photoperiod.

### Initial crosses to assess survival and fecundity

**Fig 2:**
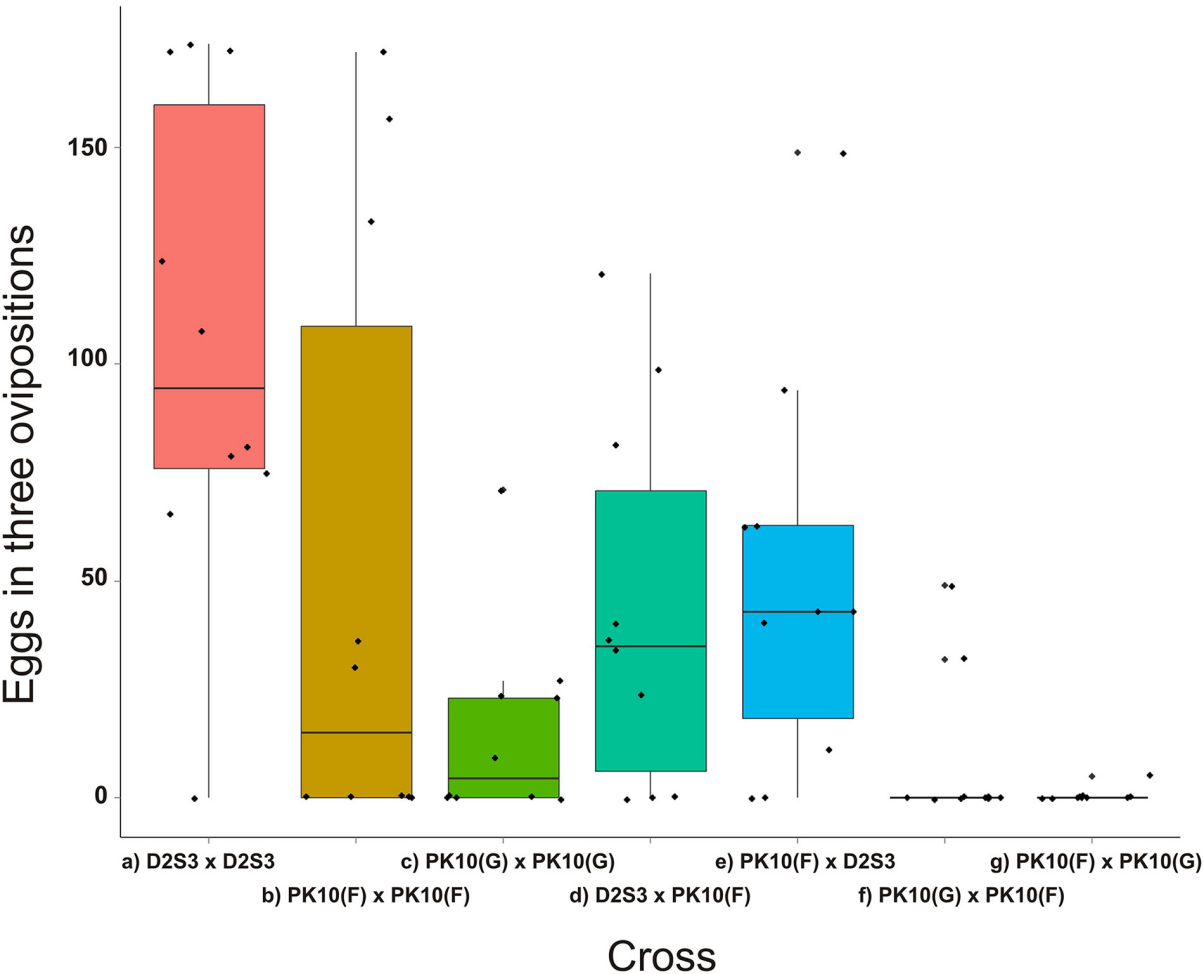
A boxplot of the total numbers of eggs obtained in three ovipositions from the 10 pairs of P_1_ adults. There were 7 parental combinations: a) female x male D2S3, b) female x male PK10(F), c) female x male PK10(G), d) D2S3 x PK10(F), e) PK10(F) x D2S3, f) PK10(G) x PK10(F), and g) PK10(F) x PK10(G). A boxplot extends from the first quartile to the third quartile and contains a horizontal line indicating the median of the data set. Two vertical lines extend from the top and bottom of the box. The bottom line extends from first quartile to the smallest value in the data set, and the top line extends from the third quartile to the largest value.

For the D2S3 (*Aaa*) by PK10 (*SenAae*) crosses, 10 pairs of virgin P_1_ parents were each given the opportunity to mate in half liter cartons, blood fed on the senior author’s arm on days 3, 5 and 8 post-eclosion, and provided on day 5 with moist paper towels on which to oviposit. Cartons were checked every day for eggs, and if present these were collected and counted until three ovipositions had occurred or 10 days had passed since the day 8 blood feeding. Lack of normality was visually evident (black dots in Fig 2) and so egg counts were log transformed (Log_10_ (eggs +1)). Transformed counts were compared using a General Linear Model (glm()) and contrasts in R 3.1.0 [37].

**Fig 3:**
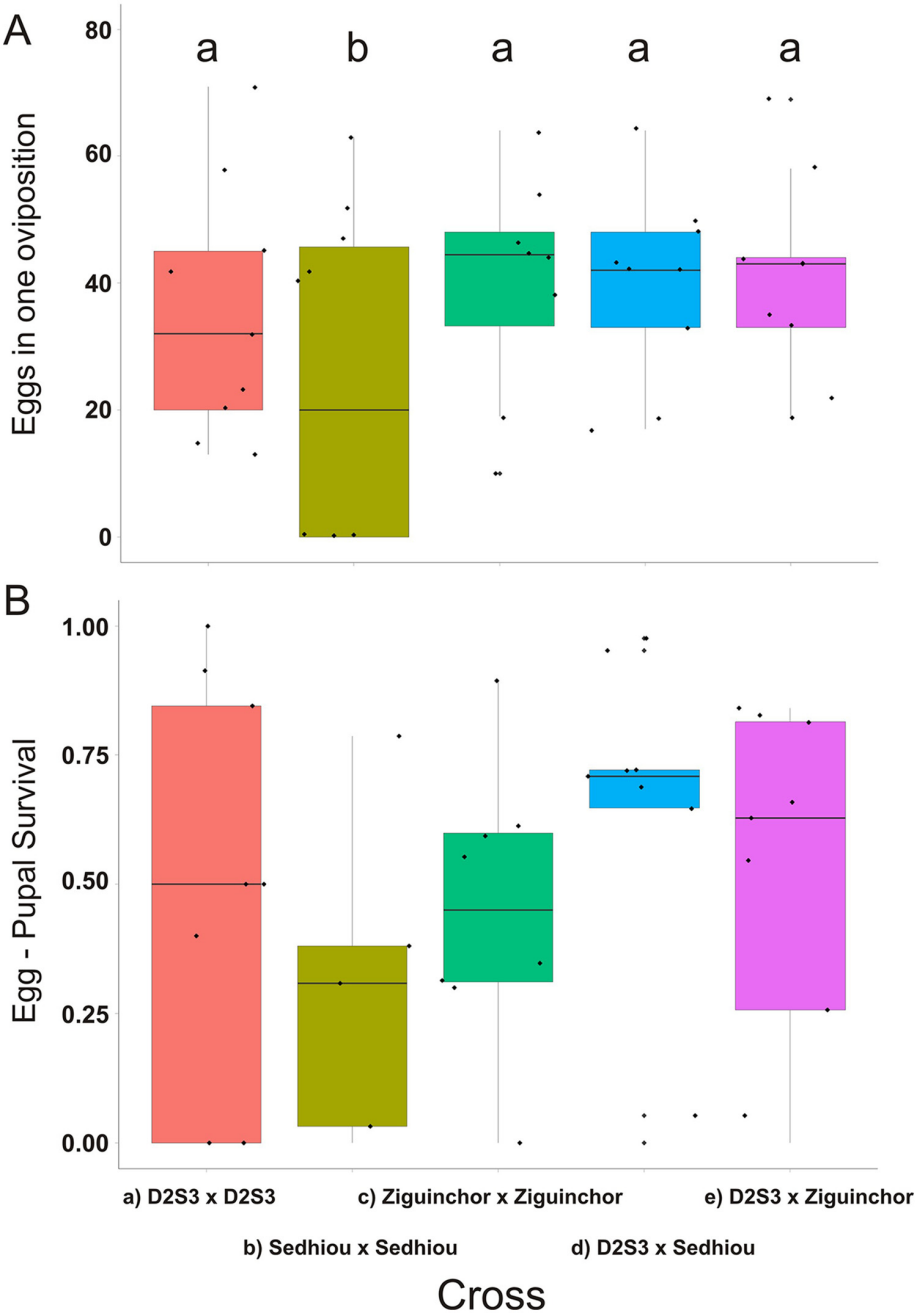
(A) Boxplot of the number of eggs produced and (B) egg to pupal survival in D2S3, Ziguinchor(F) and Sedhiou (F) families and when Ziguinchor(F) and Sedhiou (F) males were crossed to D2S3. Boxplots with homogeneous distributions appear under the same letter “a” or “b.”

The same statistical procedures were followed for the Sedhiou (F) and Ziguinchor (F) families, except eggs were only collected once, 3–4 days after blood-feeding, before adults were collected and frozen for anticipated eventual use in QTL mapping. Since our goal was to generate large F_2_ families for QTL mapping and Senegal females generally produce far fewer eggs than the D2S3 or ROCK females, F_1_ intercrosses were only made in one direction. The data from the Sedhiou (F) and Ziguinchor (F) families (black dots in Fig 3) were normally distributed (as determined by shapiro.test in R) and so Fisher’s least significant difference with Bonferroni’s correction for multiple comparisons were calculated in R 3.1.0. using pairwise t-tests on untransformed egg counts among crosses and a Bonferroni corrected probability (R command: pairwise.t.test (Eggs, Cross, p.adj = "bonferroni").

### Prezygotic barriers

Individual pairs of newly emerged virgin adults were placed together in half liter cartons and maintained with sugar water for 10 days. Females were blood fed on days 3 and 5. Following oviposition or no later than day 10, females were knocked down with trimethylamine and the spermathecae were dissected in phosphate buffered saline (PBS) from the tip of the abdomen, transferred to a glass slide, a coverslip was gently placed over them and the three spermathecae were inspected at 40X under a compound microscope to check for motile sperm. At the same time, the ovaries were inspected for abnormal development (e.g. no vitellogenin accumulation or hypotrophic ovarioles).

Many possible reasons exist for the inability of *SenAae* to survive and reproduce in the laboratory relative to long established ROCK and D2S3 lab strains. Cursory comparisons were made of ROCK, D2S3 and *SenAae* larvae and pupae grown alongside one another and fed the same larval food. While mortality occurred in larvae in all containers, there was no obvious differential mortality in *SenAae*. Different types of food were offered to the larvae and this did not differentially affect survival in *SenAae* larvae and pupae. Different oviposition container sizes, shapes and colors were tried. Coconut milk (5% (v/v)) was tried in place of water in the oviposition containers because of the abundance of *Ae*. *aegypti* larvae in *Saba senegalensis* husks [35] in forested regions. None of these conditions affected oviposition rates or larval survival. Therefore the possibility of post-zygotic reproductive incompatibility within and among *SenAae* was considered. More specifically the possibility was considered that *SenAae* collections consist of mixtures of cryptic taxa that are reproductively isolated from one another and from *Aaa*.

### Postzygotic barriers

Haldane’s rule predicts that in fully or partially reproductively isolated species with heterogametic sex chromosomes, the heterogametic sex will be preferentially sterile and inviable in interspecific crosses. Backcrosses were performed to test for postzygotic isolation patterns consistent with Haldane’s rule [38] modified for species, like *Aedes*, that have an autosomal sex determining locus (SDL) [39]. More specifically, in crosses between the ROCK *Aaa* strain and *SenAae* strains, crosses involving hybrid F_1_ males were expected to have higher sterility but crosses involving hybrid F_1_ females to be unaffected. Egg hatch was predicted to be low when a female mates to a hybrid F_1_ male but hatch was predicted to be average when a male mates to a hybrid F_1_ female.

F_1_ mosquitoes were generated by placing 50 virgin ROCK females in a 30 cm x 30 cm x 30 cm cage with 50 PK10 (F) males, these were blood fed once. Reciprocal crosses were made in a second cage and blood fed once. Control crosses were generated by placing a) 50 virgin ROCK females with 50 virgin ROCK males and f) 50 virgin PK10 (F) females with 50 virgin PK10 (F) males. Eight backcrosses were then made with the F_1_ progeny. These were: b) ROCK x (ROCK x PK10 F_1_), c) ROCK x (PK10 x ROCK F_1_), d) (ROCK x PK10 F_1_) x ROCK, e) (PK10 x ROCK F_1_) x ROCK, g) ROCK x (ROCK x PK10 F_1_), h) ROCK x (PK10 x ROCK F_1_), i) (ROCK x PK10 F_1_) x PK10, j) (PK10 x ROCK F_1_) x PK10. For each of the ten crossing types, 40 families were set up in half liter cartons. Eggs and then pupae arising from each of these crossing types were counted in each of the ten crossing types. Egg counts in most crosses were zero-inflated (see S1 Table), so zero-inflated negative binomial (ZINB) regression in R 3.1.0 was used with the zeroinfl function in the pscl package. In addition glm() in R 3.1.0 was used to compare log_10_(eggs) including only those females that laid eggs. The proportions of eggs surviving to pupae were compared using glm() in R 3.1.0. Eggs that failed to hatch were checked under a dissecting scope to determine if they were desiccated (collapsed). If not they were then soaked in fresh Trpis bleach [40] for 4 hours and then observed at 20–40X under a compound microscope. No evidence of embryo formation (e.g. segmentation) was observed in these unhatched eggs.

### Chromosome rearrangements

To evaluate the possibility that chromosome rearrangements occur within SenAae and between *SenAae* and *Aaa*, chromosomes were measured, stained to identify heterochromatic regions and analysed with Fluorescent *In Situ* Hybridization (FISH) with markers of known position in the *Aaa* genome to test for chromosome rearrangements. In addition, linkage distances in cM were estimated between the sex determining locus and white eye on chromosome 1 and compared among collections.

Chromosomal preparations were made from 4^th^ instar larvae [41]. Chromosomes were stained with 1 μM YOYO-1 iodide solution (Invitrogen Corporation, Carlsbad, CA, USA) in 1x PBS and enclosed under anti-fade Prolong Gold reagent (Invitrogen Corporation, Carlsbad, CA, USA). Chromosome lengths were measured using Zen 2009 light edition software (http://www.zeiss.de). Based on these measurements, chromosome indexes (the percentage of each arm in the total chromosome length) and centromeric indices (length of the short arm p relative to the total length of the chromosome) were calculated and *P*-values were determined [42].

Chromosomes were stained with a DAPI Prolog Gold Reagent (Invitrogen Corporation, Carlsbad, CA, USA) and chromomycin A3 (Sigma-Aldrich Corporation, St. Louis, MO, USA) to identify differences in heterochromatic regions between strains. Fluorescent staining was performed with DAPI/Chromomycin A3 [43]. FISH was performed as described previously [44]. BAC clones with known locations on chromosomes of the IB12 strain [45] were utilized. Unspecific hybridization was blocked by using unlabeled repetitive DNA (C_0_t3) fractions.

The linkage distance of the white-eye marker [46] at 20 cM and the sex determining locus (SDL) at 34 cM [47] on chromosome 1 was estimated as a quick and inexpensive way to test for rearrangements on chromosome 1. Forty P_1_ families were set up in each of four SenAae collections (Mont Rolland, Bignona, PK10 and Goudiry (Fig 1) and in 20 families from an *Aaa* collection from Merida, Mexico (Fig 1).

## Results

### Survival and fecundity in crosses within and between Senegal collections

To assess survival and fecundity within *SenAae* and between *SenAae* and *Aaa* collections, a series of crosses were set up that involved the laboratory strain D2S3 and PK-10 *SenAae* (Fig 1) that either had scales on the first abdominal tergite, PK10(G), or had no scales, PK10(F). There were 7 parental combinations (Fig 2): a) female x male D2S3, b) female x male PK10(F), c) female x male PK10(G), d) D2S3 x PK10(F), e) PK10(F) x D2S3, f) PK10(G) x PK10(F), and g) PK10(F) x PK10(G). Table 2 lists the hypothesis being addressed by each contrast in the General Linear Model. The numbers of eggs produced in three ovipositions ranged from very high in the D2S3 family to nearly zero in crosses between PK10(F) and PK10(G) parents (Fig 2). D2S3 families produced twice the number of eggs as PK10(F) and ~7x more than PK10(G) families and these differences were significant (Table 2—Contrast 1). The numbers of eggs produced in PK10(F) and PK10(G) families were not significantly different (Table 2—Contrast 2). Reciprocal crosses between D2S3 and PK10(F) produced equal numbers of eggs (Table 2—Contrast 3) but half the number produced by D2S3 (Table 2—Contrast 4). There was a large and significant drop in fecundity in the PK10(F) family as compared to PK10(F) x PK10(G) crosses (Table 2—Contrast 5) and in the PK10(G) family as compared to PK10(G) x PK10(F) crosses (Table 2—Contrast 6) is undocumented. PK10(F) x PK10(G) reciprocal crosses had similarly low fecundity (Table 2—Contrast 7). These experiments were terminated prior to intercrossing F_1_ siblings because most crosses yielded too few eggs to continue onto the F_2_.

**Table 2:** Pairwise t-tests to compare log_10_ (eggs+1) among crossing types in Fig 2.

Hypothesis	Estimate	Std.Err	t–value	Pr(>|t|)	Answer
1. *Aaa* fecundity > *SenAae* (a vs. b&c)?	0.821	0.156	5.244	<0.0001***	Yes
2. *SenAae* with different scaling differ in fecundity (b vs. c)?	0.128	0.168	0.760	0.4502	No
3. *Aaa x SenAae* reciprocal crosses differ in fecundity (d vs. e)?	-0.081	0.168	-0.480	0.6332	No
4. *Aaa* families > fecundity *Aaa x SenAae* crosses (a vs. d)?	-0.366	0.142	-2.570	0.0125*	Yes
5. *SenAae* (F) crosses < fecundity than *SenAae* (Fx G) (b vs. g)crosses?	-0.850	0.221	-3.839	0.0003***	Yes
6. *SenAae* (G) crosses < fecundity than *SenAae* (G x F) (c vs. f) crosses?	-0.606	0.221	-2.737	0.0080**	Yes
7. *SenAae* (Gx F) differ in fecundity from *SenAae* (F G) families (f vs.g)?	-0.172	0.239	-0.722	0.4732	No

To assess fecundity in F_1_ siblings, another series of F_1_ intercrosses were established with *SenAae* from Sedhiou and Ziguinchor from the Casamance area of southern Senegal (Fig 1) to determine whether the results seen in the first experiment were unique to PK10. These collections also had a large proportion of *SenAae* without scales on the first abdominal tergite. Ziguinchor(F) and Sedhiou (F) were crossed with one another and to D2S3. Egg to pupal survival in P_1_ and F_1_ eggs were recorded as was F_2_ pupal-to-adult survival. Fig 3A shows the number of eggs produced by a) D2S3, b) Sedhiou(F), and c) Ziguinchor (F) families, as well as d) D2S3 females crossed to Sedhiou (F) males and e) D2S3 females crossed to Ziguinchor (F). All but the Sedhiou families produced similar numbers of eggs. There was wide variation in pupal survival (pupae/eggs) within all five crossing types but all were statistically homogeneous (Fig 3B).

**Fig 4:**
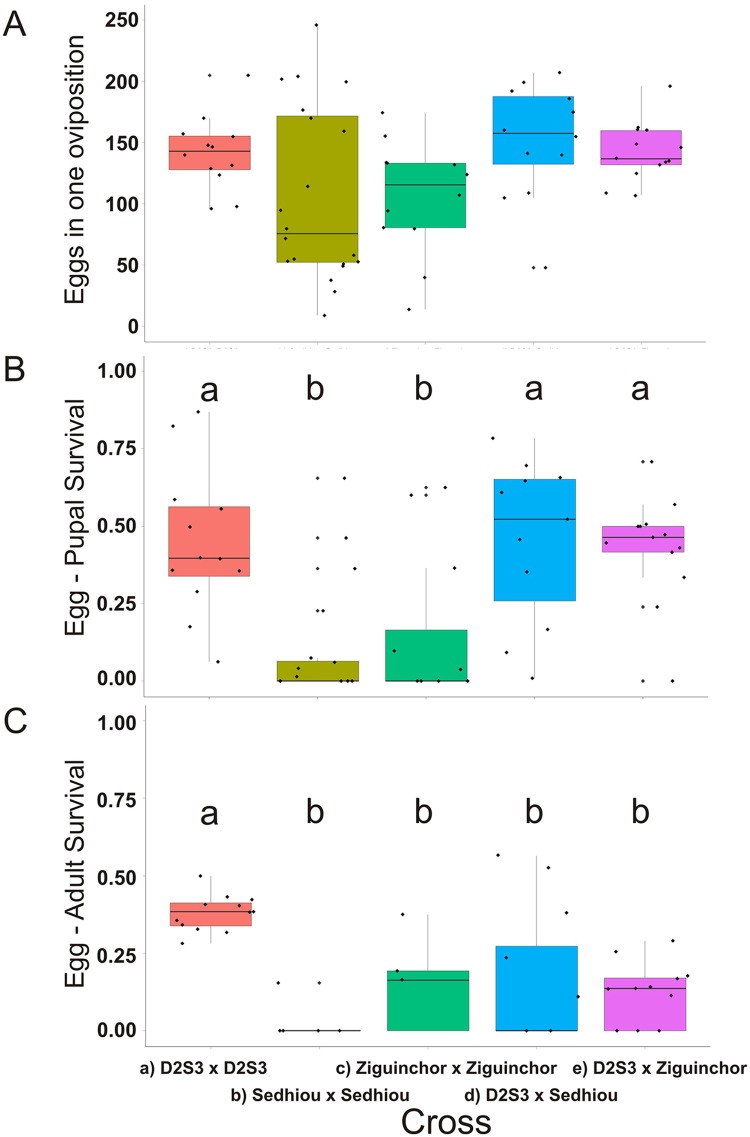
(A) Boxplot of the number of eggs produced, (B) egg to pupal survival and (C) egg to adult survival in D2S3, Ziguinchor(F) and Sedhiou (F) families and when Ziguinchor(F) and Sedhiou (F) males were crossed to D2S3. Boxplots with homogeneous distributions appear under the same letter “a” or “b.”

The number of eggs produced when intercrossing the F_1_ siblings to generate F_2_ families in each of the five crossing types was also recorded (Fig 4A). The number of eggs produced was statistically homogeneous among the five crossing types. Pupal and adult survival in each of the five crossing types was also measured (Fig 4B and 4C). Pupal survival was homogeneous among D2S3 families, D2S3 x Sedhiou(F) and D2S3 x Ziguinchor(F). However pupal and adult survival in Sedhiou(F) and Ziguinchor(F) families were significantly lower. Thus while all five types of crosses produced similar numbers of F_1_ eggs (Fig 3A) and had similar F1 pupal survival rates (Fig 3B) survival of these eggs to F_2_ adults was only 0.02–0.16 in Sedhiou(F) and Ziguinchor(F) families and in the D2S3 x Sedhiou(F) and D2S3 x Ziguinchor(F) hybrids. *SenAae* exhibit poor egg-adult survival in the laboratory as do hybrids of these strains when mated to long established lab colonies. To determine whether the results in Figs 2–4 were specific to D2S3, the experiment was repeated with the ROCK strain and Sedhiou(F). Similar results were obtained and no further attempts were made to construct F_1_ intercross families for QTL mapping.

### Prezygotic barriers

**Fig 5:**
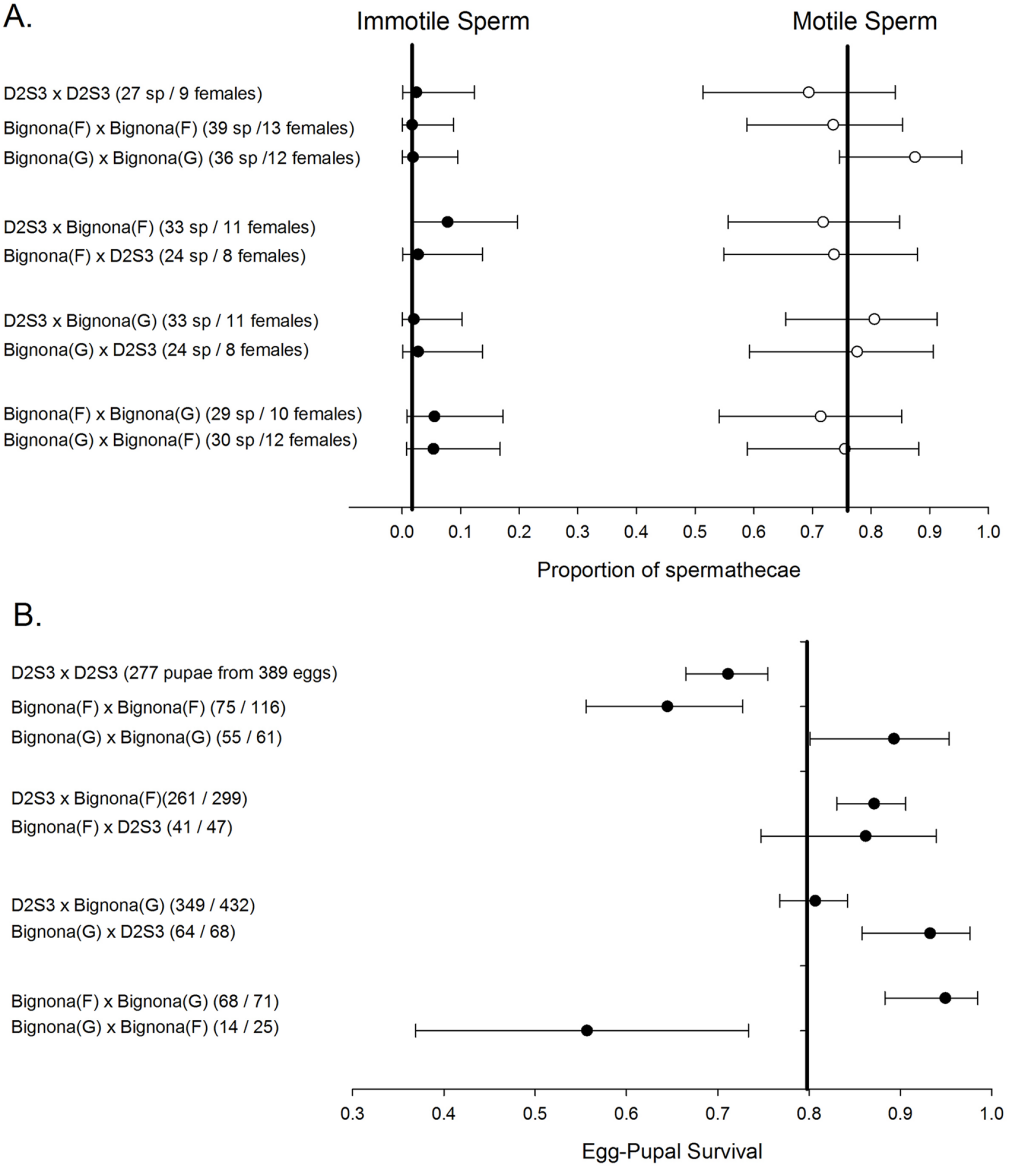
**A) Post-zygotic reproductive incompatibility within and among *SenAae* collections.** Boxplots in each of the ten crossing types of the numbers of eggs produced. B) Post-zygotic reproductive incompatibility within and among *SenAae* collections. Boxplots in each of the ten crossing types of the egg-pupal survival.

A series of crosses were performed next to test for prezygotic barriers to gene flow among *SenAae*. Eggs from the February 2010 collected *SenAae* PK10(F), Sedhiou(F), and Ziguinchor (F) experienced large-scale hatch failures even after only 2–3 weeks of storage following oviposition. A newly collected July 2010 *SenAae* from Bignona (Fig 1) from which separate G and F strains could be selected was used instead. In none of the 9 parental crosses were females uninseminated and on average 77% of spermathecae contained motile sperm (Fig 5A). Furthermore there were very few females in which sperm were immotile. Ovarian development in all cases appeared to be normal. F_1_ egg-to-pupal survival was monitored in all of these females and was uniform among all crosses except for the D2S3 families, Bignona(F) intercrosses and the Bignona(G) x Bignona(F) cross (Fig 5B).

A second set of experiments were set up to test for evidence of assortative mating using the recessive white eye allele [46] as a marker for *Aaa*. Fifty Higgs White Eye (HWE) females were located in a cage with 25 HWE males and 25 Richard Toll (F) males to test for assortative mating by *Aaa* females. Of 44 families generated, 24 were white eye, not significantly more than the 22 expected if there was no discrimination (χ^2^_[1 d.f]_ = 0.36. P = 0.55). To test for assortative mating by Richard Toll(F) females, again 50 females were located in a cage with 25 HWE males and 25 Richard Toll(F) males. All F_1_ offspring were wild type and offspring adults were backcrossed to HWE adults to assess their genotypes. Of 31 backcross (BC) families, 17 had white eye offspring, not significantly more than the 15 expected if there was no discrimination (χ^2^_[1 d.f]_ = 0.29. P = 0.59). Mating between Richard Toll (F) and HWE did not appear to be assortative. This experiment was repeated but using PK10(F) (2011) choosing between 25 HWE and 25 Richard Toll (F) males. Of 36 families generated, 24 were white eye, slightly more than the 18 expected if there was no discrimination (χ^2^_[1 d.f]_ = 4.0. P = 0.046). All surviving F_1_ families were wild type and offspring were backcrossed to HWE adults to assess their genotypes but this only produced 2 BC families. This experiment was repeated with Goudiry (F). Of 20 HWE families, 6 were white eye, not significantly fewer than the expected 10 if there was no discrimination (χ^2^_[1 d.f]_ = 3.20. P = 0.074). When Goudiry (F) females were located in a cage with equal numbers of HWE and Goudiry males, only 6 BC families survived. These experiments did not reveal any obvious prezygotic barriers to mating among *Aaa* and *SenAae*.

### Postzygotic reproductive isolation

Haldane’s rule predicts that in fully or partially reproductively isolated species with heterogametic sex chromosomes, the heterogametic sex will be preferentially sterile and inviable in interspecific crosses. Presgraves and Orr [39] tested Haldane’s rule in *Aedes* mosquitoes, which contain homomorphic sex chromosomes that contain a sex determining locus and a dominant male allele, and determined that mosquitoes in the genus *Aedes* follow Haldane’s rule for sterility, but not for inviability. To test for patterns of sterility consistent with Presgraves and Orr [39] caveat to Haldane's rule, Ten crosses were made: A. 50 virgin ROCK females mated with 50 virgin ROCK males, B. ROCK x (ROCK x PK10 F_1_), C. ROCK x (PK10 x ROCK F_1_), D. (ROCK x PK10 F_1_) x ROCK, E. (PK10 x ROCK F_1_) x ROCK, F. 50 virgin PK10(F) females mated with 50 virgin PK10(F) males, G. PK10 x (ROCK x PK10 F_1_), H. PK10 x (PK10 x ROCK F_1_), I. (ROCK x PK10 F_1_) x PK10, J. (PK10 x ROCK F_1_) x PK10.

Haldane’s rule could not be tested in the strictest sense because no markers exist to distinguish reproductively isolated taxa in *SenAae*. Instead trends consistent with Haldane’s rule in *SenAae* were tested for assuming collections contain both reproductively compatible and incompatible individuals. More specifically, in crosses between the ROCK *Aaa* strain and *SenAae* strains, crosses involving hybrid F_1_ males were expected to have higher sterility but crosses involving hybrid F_1_ females were expected to be unaffected. Egg hatch is predicted to be low when a female mates to a hybrid F_1_ male but hatch is predicted to be average when a male mates to a hybrid F_1_ female.

**Fig 6:**
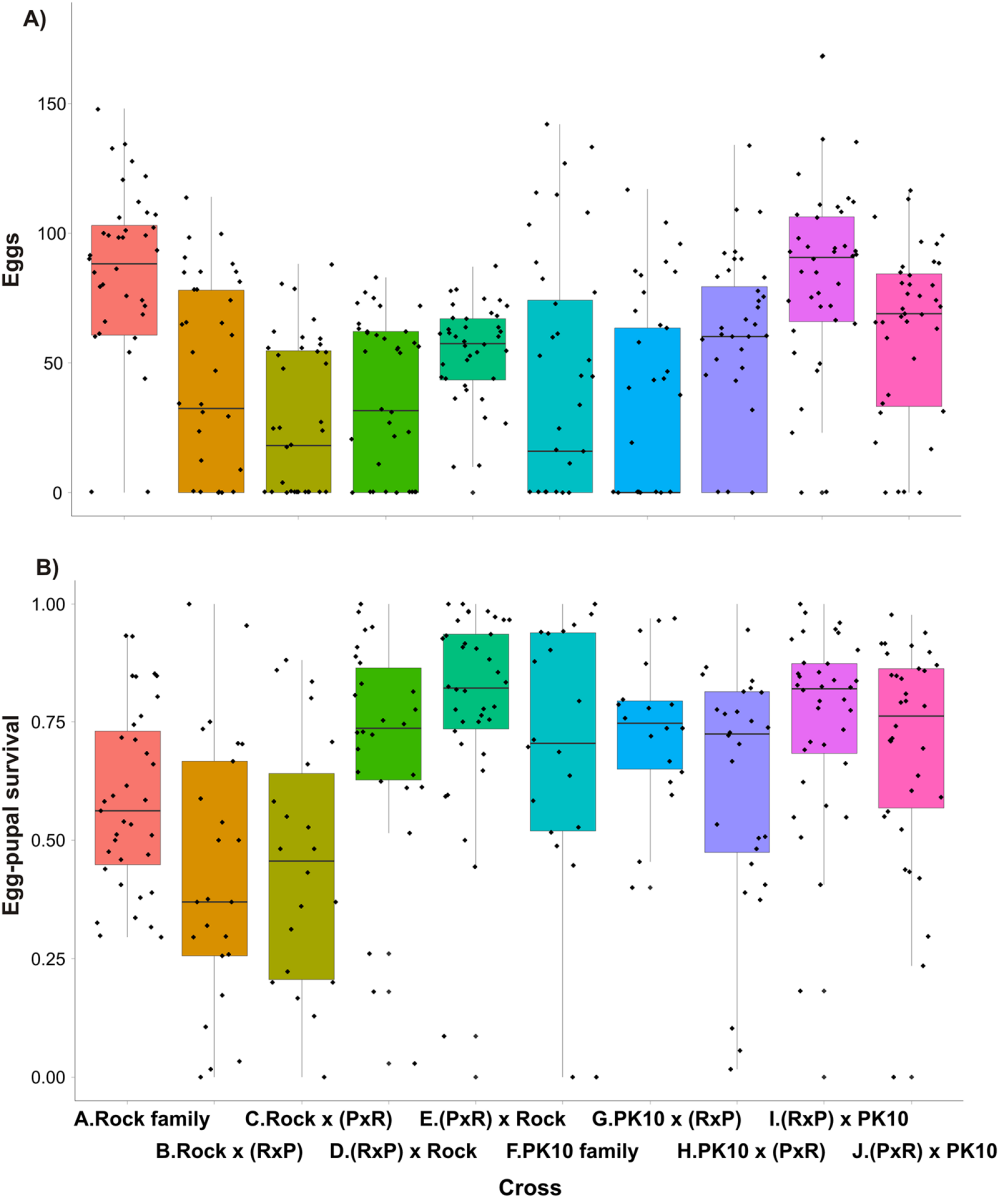
Prezygotic barriers to gene flow within and among D2S3, Bignona (G) and Bignona (F) strains. Pairs of newly emerged virgin adults were placed together in half liter cartons and maintained with sugar water for 10 days. Females were blood fed on days 3 and 5. Following oviposition, spermathecae were inspected at 40X under a compound microscope to check for motile sperm in all three spermathecae. (A) The number of spermathecae (sp) checked per female and indicates that in no case were females uninseminated and on average 77% of spermathecae contained motile sperm. (B) The number of F_1_ eggs that survived to pupae. The proportion surviving is graphed alongside the Bayesian 95% Highest Density Interval (HDI). Survival rates were uniform among all crosses except for the D2S3 and Bignona(F) intercrosses and the Bignona(G) x Bignona(F) cross.

The number of eggs/female (Fig 6A) and egg-pupal survival (Fig 6B) in each of the ten crossing types (a–j) were analyzed. Statistical analyses were performed of the proportion of females ovipositing (S1 Table), numbers of eggs produced by all females (S2 Table), numbers of eggs produced by ovipositing females (S3 Table) and egg-pupal survival (S4 Table). The qualitative results with respect to Haldane’s rule are summarized in Tables 3 and 4. The first four contrasts in Table 3A indicate that offspring of crosses involving ROCK/PK10 hybrid F_1_ males had lower egg-pupal survival than offspring of ROCK families as predicted by Haldane’s rule if Aaa and SenAae are separate taxa. The next four contrasts show that offspring of crosses involving ROCK/PK10 hybrid F_1_ males had the same low egg-pupal survival as offspring of PK10 families. This pattern suggests that *SenAae* consists of a single taxon.

**Table 3:** Qualitative summary of Presgraves and Orr’s predictions that hybrid F_1_ males will have higher sterility (measured as low egg-pupal survival in the offspring of hybrid mated females).

Contrast	A) Did the offspring of crosses involving hybrid F_1_ males have lower egg-pupal survival?		
1	a) ROCK x ROCK	b) ROCK x (RxP)	Yes
2	a) ROCK x ROCK	c) ROCK x (PxR)	Yes
5	b) ROCK x (RxP)	d) (RxP) x ROCK	Yes
6	c) ROCK x (PxR)	e) (PxR) x ROCK	Yes
7	f) PK10 x PK10	g) PK10 x (RxP)	No
8	f) PK10 x PK10	h) PK10 x (PxR)	No
11	g) PK10 x (RxP)	i) (RxP) x PK10	No
12	h) PK10 x (PxR)	j) (PxR) x PK10	No
Contrast	B) Did the offspring of crosses involving hybrid F_1_ females have homogeneous egg-pupal survival?		
3	a) ROCK x ROCK	d) (RxP) x ROCK	No
4	a) ROCK x ROCK	e) (PxR) x ROCK	No
5	b) ROCK x (RxP)	d) (RxP) x ROCK	No
6	c) ROCK x (PxR)	e) (PxR) x ROCK	No
9	f) PK10 x PK10	i) (RxP) x PK10	Yes
10	f) PK10 x PK10	j) (PxR) x PK10	Yes
11	g) PK10 x (RxP)	i) (RxP) x PK10	Yes
12	h) PK10 x (PxR)	j) (PxR) x PK10	Yes

**Table 4:** Qualitative summary of Presgraves and Orr’s predictions that the fecundity of crosses involving hybrid F_1_ females will be unaffected.

Contrast	A) Was the fecundity of crosses involving hybrid F_1_ males unaffected?					
			S1 Table	S2 Table	S3 Table	All considered
1	a) ROCK x ROCK	b) ROCK x (RxP)	No	No	No	No
2	a) ROCK x ROCK	c) ROCK x (PxR)	No	No	No	No
5	b) ROCK x (RxP)	d) (RxP) x ROCK	Yes	Yes	Yes	Yes
6	c) ROCK x (PxR)	e) (PxR) x ROCK	No	No	Yes	No/Yes
7	f) PK10 x PK10	g) PK10 x (RxP)	Yes	Yes	Yes	Yes
8	f) PK10 x PK10	h) PK10 x (PxR)	Yes	Yes	Yes	Yes
11	g) PK10 x (RxP)	i) (RxP) x PK10	No	No	No	No
12	h) PK10 x (PxR)	j) (PxR) x PK10	Yes	Yes	Yes	Yes
	B) Was the fecundity of crosses involving hybrid F_1_ females unaffected?					
			S1 Table	S2 Table	S3 Table	All considered
3	a) ROCK x ROCK	d) (RxP) x ROCK	Yes	no	No	No/Yes
4	a) ROCK x ROCK	e) (PxR) x ROCK	Yes	yes	No	No/Yes
5	b) ROCK x (RxP)	d) (RxP) x ROCK	Yes	yes	Yes	Yes
6	c) ROCK x (PxR)	e) (PxR) x ROCK	No	no	Yes	No/Yes
9	f) PK10 x PK10	i) (RxP) x PK10	No	No	No	No
10	f) PK10 x PK10	j) (PxR) x PK10	No	No	Yes	No/Yes
11	g) PK10 x (RxP)	i) (RxP) x PK10	No	No	No	No
12	h) PK10 x (PxR)	j) (PxR) x PK10	Yes	Yes	Yes	Yes

The first four contrasts in Table 3B show that offspring of crosses involving ROCK/PK10 hybrid F_1_ females have greater egg-pupal survival than offspring of ROCK families. This pattern is inconsistent with Haldane’s predictions that egg-pupal survival should be unaffected in crosses involving hybrid females. The next four contrasts show that offspring of crosses involving ROCK/PK10 hybrid F_1_ females had the same egg-pupal survival as offspring of PK10 families. This pattern also suggests that *SenAae* consists of a single taxon.

The first four contrasts in Table 4A are equivocal. The fecundity of crosses involving hybrid F_1_ males was reduced in the first two contrasts and unaffected in the next two. This pattern is therefore inconclusive with respect to Haldane’s predictions. The next four contrasts are similarly inconclusive with crosses involving ROCK/PK10 hybrid F_1_ males having the same fecundity as offspring of PK10 families in three of the four contrasts. Table 4B is also inconclusive.

In summary, patterns of egg-pupal survival are only partially consistent with Haldane’s predictions if *Aaa* and *SenAae* were fully or partially reproductively isolated species. But patterns are consistent with *SenAae* consisting of a single taxon. In contrast, fecundity patterns are inconclusive with Haldane’s predictions because most of the reduced fecundity was caused by a lack of oviposition by mated females rather than reduced survival in her offspring.

### Cytogenetic analysis of chromosomes in four *SenAae* strains

**Fig 7:**
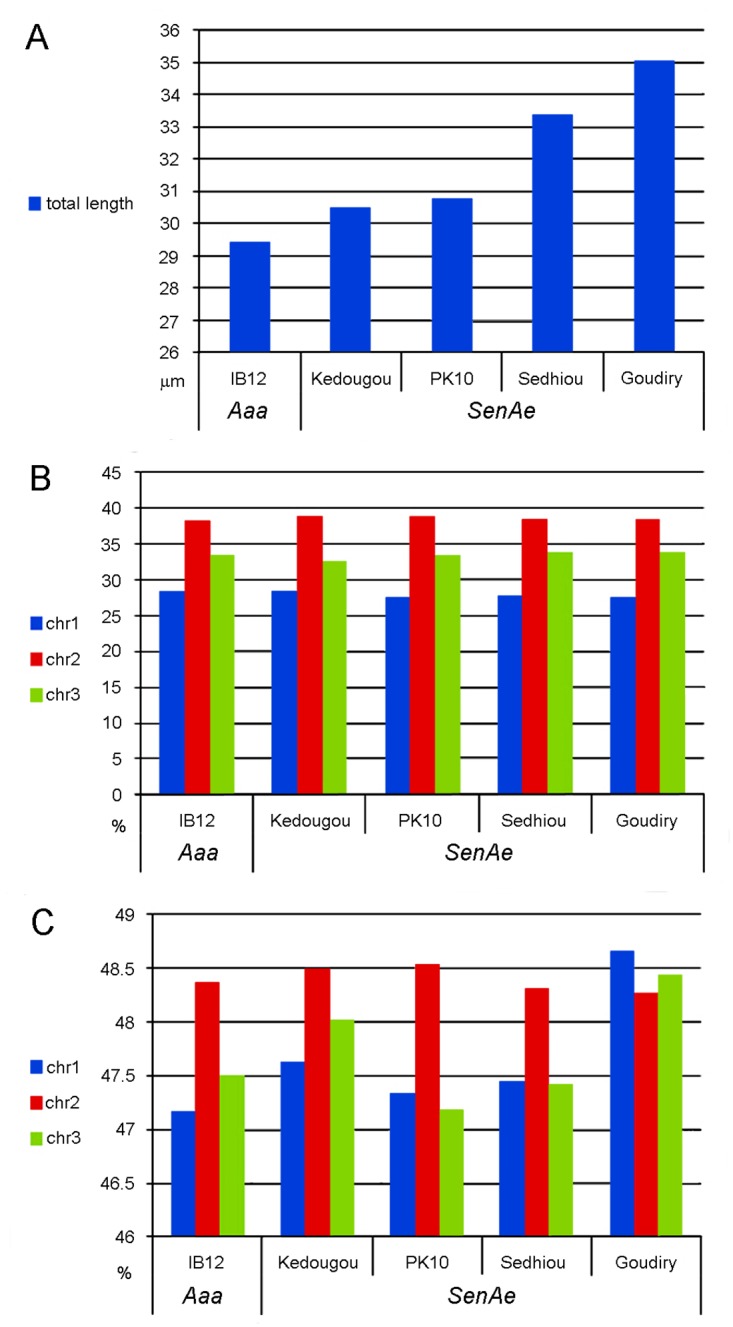
The chromosome measurements in IB12 strain of *Aaa* and 4 African strains of *SenAae*. Total chromosome length (**A**), relative lengths of 3 chromosomes (**B**) and centromeric indexes of 3 chromosomes (**C**) were compared.

A reliable protocol to prepare somatic chromosomes of *Ae*. *aegypti* from leg imaginal discs in fourth instar larvae has been developed [41] and has allowed the physical mapping of scaffolds onto *Ae*. *aegypti* chromosomes using FISH [48]. In this study this approach was utilized for cytogenetic analysis of four of the *SenAae* strains: Kedougou, Sedhiou, PK10, and Goudiry (Fig 1). As a control the IB12 strain of *Aaa* was used because it was the genome that had been sequenced [33] and physically mapped [48]. To determine the presence of chromosomal rearrangements in *SenAae* strains, basic cytogenetic analyses (chromosome measurements and differential staining) and FISH experiments were performed. Although all *SenAae* strains have the typical *Ae*. *aegypti* karyotype of three pairs of metacentric chromosomes [49], basic chromosome analysis revealed differences between them. Sedhiou and Goudiry mosquitoes have significantly longer chromosomes (*P* = 0.0065 and <0.0001 for Sedhiou and Goudiry strains, respectively) as compared to IB12 strain of *Aaa* (Fig 7A). This is probably due to differences in repetitive element content. Relative chromosome lengths were identical suggesting the absence of inter-chromosomal rearrangements such as translocations (Fig 7B). The Goudiry strain had significant differences from other strains in centromeric indexes of chromosomes 1 and 3 suggesting the possibility of pericentric inversions (Fig 7C). However, the differences were only 1.5% and 1%, and *P*-values were 0.0051 and 0.0122 for chromosomes 1 and 3, respectively

**Fig 8:**
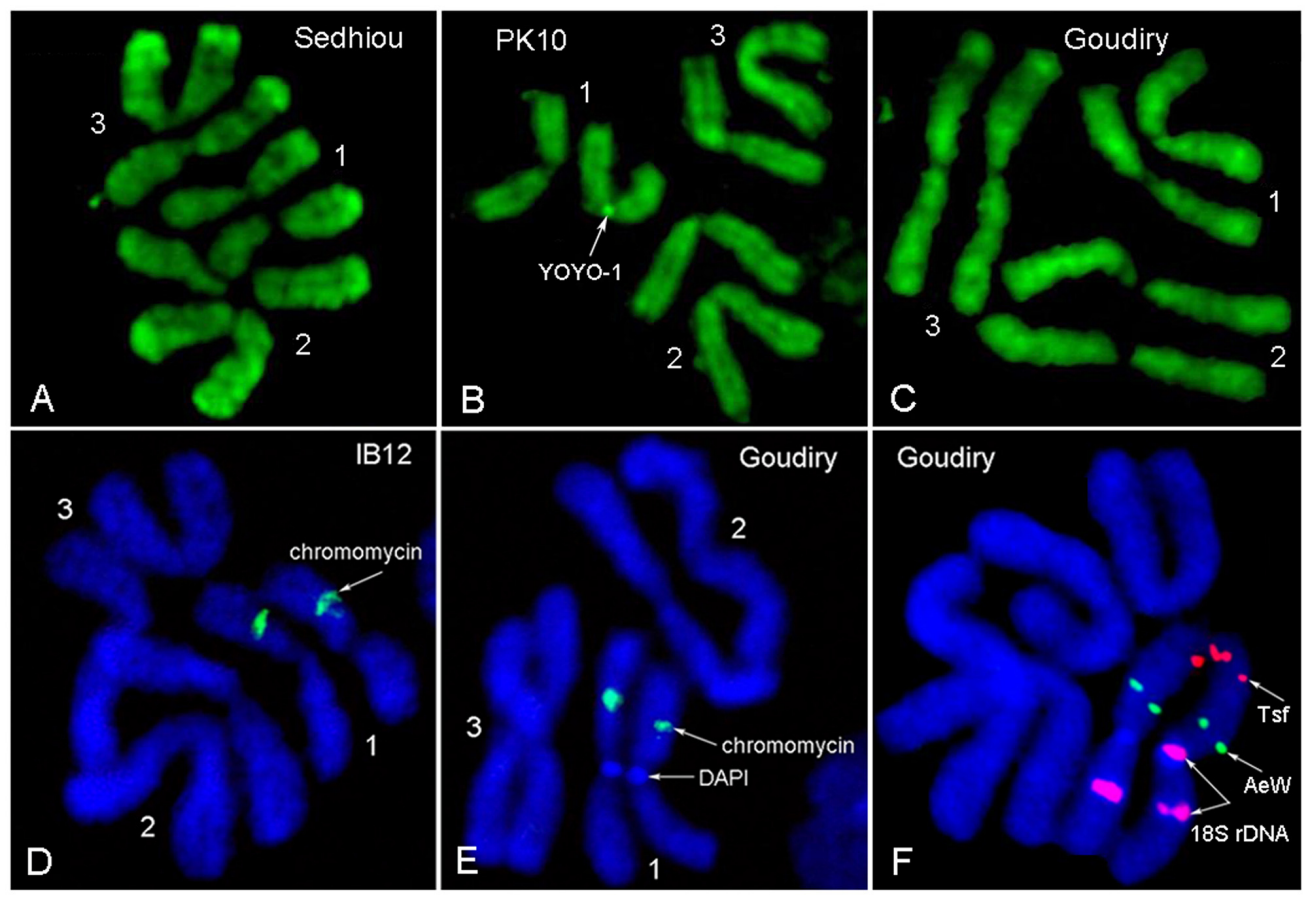
**The differences in chromosome banding patterns in IB12 and 3 African strains of *Aaf*. YOYO-1 staining (A-C) revealed presence of a dark polymorphic band in chromosome 1 in the PK10 strain (B).** Ribosomal locus in all strains was stained by chromomycin (**D,E**). Additional DAPI staining band was found in centromere of chromosome 1 in the Goudiry strain. FISH with BAC clones containing the transferrin (*tsf*) gene (red), *white eye* (AeW) (green) and *rDNA* (purple) genes demonstrated the presence of an additional ribosomal locus in this band (**F**). Note that two regions of rDNA staining exist on one of the chromosome 1 copies while a single region stains in the *Aaa* strain IB12 [48].

Polymorphisms in the amount and positions of C-positive heterochromatic bands have been previously described in natural population of *Ae*. *aegypti* [50–52]. Two fluorescent dyes were used for identification of heterochromatic regions: DAPI for staining AT-rich regions and chromomycin A3 with pretreatment with barium hydroxide for staining of GC-rich regions [53] and YOYO-1 was used to test for differential staining. These experiments indicated that chromosome patterns of *SenAae* strains are different from the patterns in *Aaa* (Fig 8). In addition to chromomycin stained (GC-rich) band, which represents the ribosomal RNA locus of all strains (Fig 8D), the Goudiry strain had DAPI stained (AT-rich) band (Fig 8E) in the centromere of chromosome 1. A FISH experiment with 18S rDNA probe has indicated the presence of an additional ribosomal locus on one of homologous chromosomes in this region (Fig 8F). PK10 and Kedougou strains had polymorphic bright YOYO-1 bands in centromeres of chromosome 1 (Fig 8B) and 3. Thus, all strains had differences in the structure of heterochromatic regions of chromosomes and suggest that the strains are genetically diverged.

**Fig 9:**
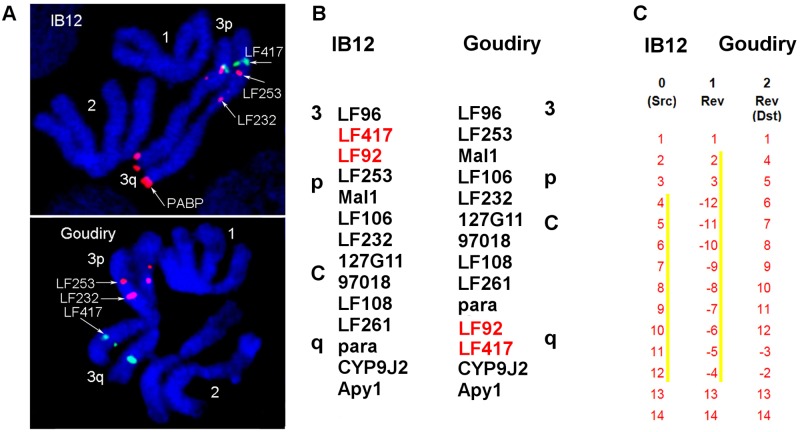
Evidence for a pericentric inversion on chromosome 3 between *SenAae* from Goudiry and IB12. The marker LF417 changes its chromosomal position from 3p to 3q arm in Goudiry (**A**). Pattern comparison of 14 markers in chromosomes between IB12 strain of *Aaa* and Goudiry strain (**B**) suggests presence of 2 large overlapping pericentric inversions in chromosome 3 or insertion on 3Q arm (**C**). BAC clones are named by genetic markers they are carrying.

**Fig 10:**
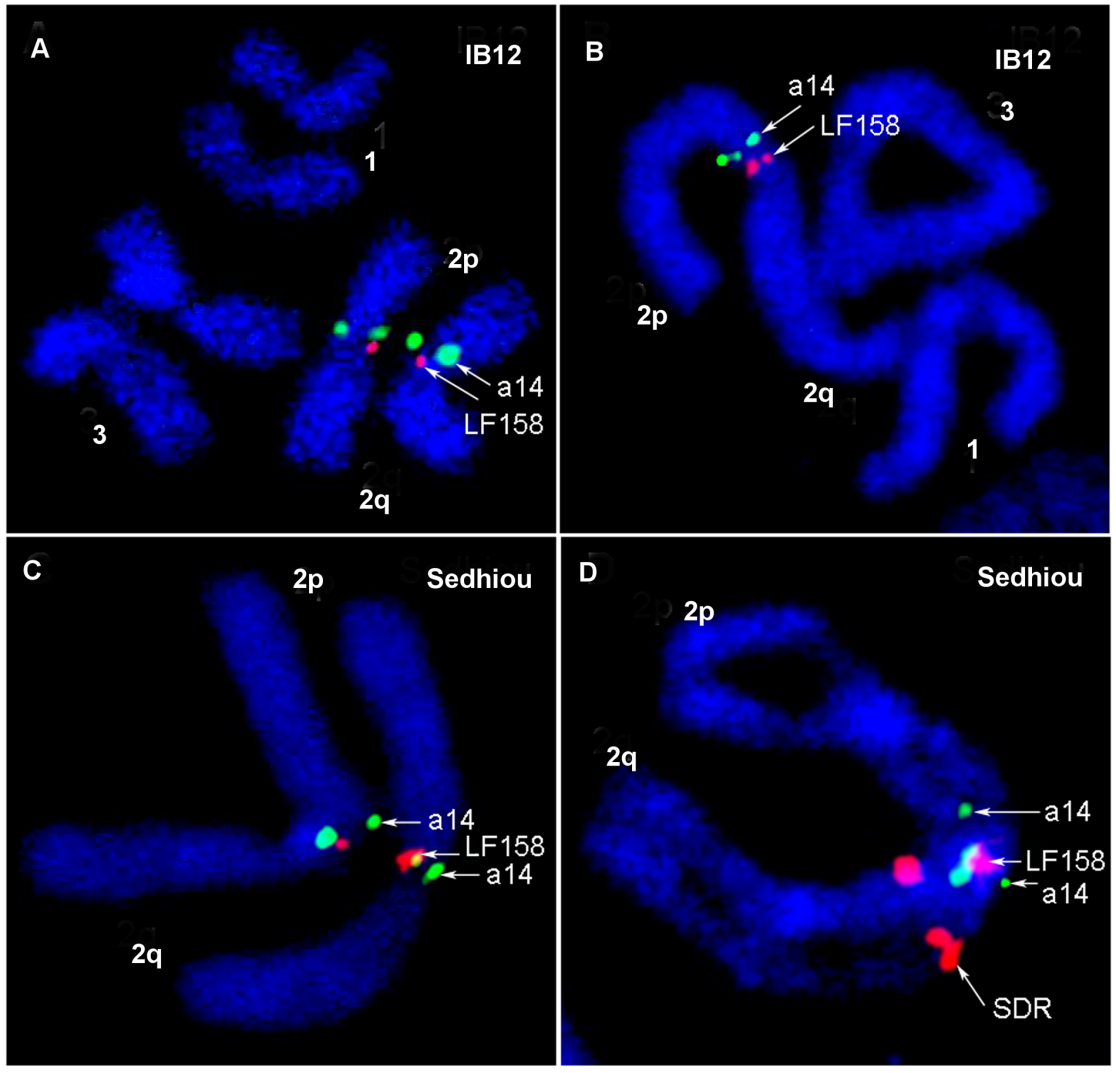
The standard (A, B) and heterozygote (C, D) arrangement of the inversion on chromosome 2 in IB12 and Sedhiou strains respectively. BAC clones are named by genetic markers they are carrying.

In addition to the basic cytogenetic analyses, 40 BAC clones previously physically mapped on *Aaa* chromosomes were used as markers for the identification of chromosome rearrangements between the two subspecies. BAC clones were hybridized to the chromosomes of Sedhiou strain using the standard FISH protocol[44]. Although the resolution of this mapping was relatively low ~30 Mb, the differences in the order of markers revealed the presence of two chromosomal rearrangements between *Aaa* and *SenAae* strains. The first rearrangement was found on chromosome 3 (Fig 9A). Markers LF417 and LF92 dramatically changed their positions from the 3p arm in *Aaa* to the 3q arm in all *SenAae* strains. Importantly, non-specific hybridization of all repetitive sequences including transposable elements was blocked in the FISH experiments by adding unlabeled C_0_t3 DNA fractions to the probe before hybridization to the chromosomes. The positions of 14 markers on chromosome 3 in the Goudiry strain (Fig 9B) were analyzed using the program Genome Rearrangements In Man and Mouse program (grimm.ucsd.edu/GRIMM/). The difference in the patterns between *Aaa* and *SenAae* strains suggests the presence of two overlapping pericentric inversions in chromosome 3 or an insertion of a large fragment into 3q arm (Fig 9C). The second rearrangement was found closely located to the centromere on the 2p arm. Positions of markers LF158 and a14 were found only in the standard arrangement in IB12 chromosomes (Fig 10A and 10B) and in the standard or standard/inverted arrangements (Fig 10C and 10D) in the Sedhiou and Goudiry strains.

**Fig 11:**
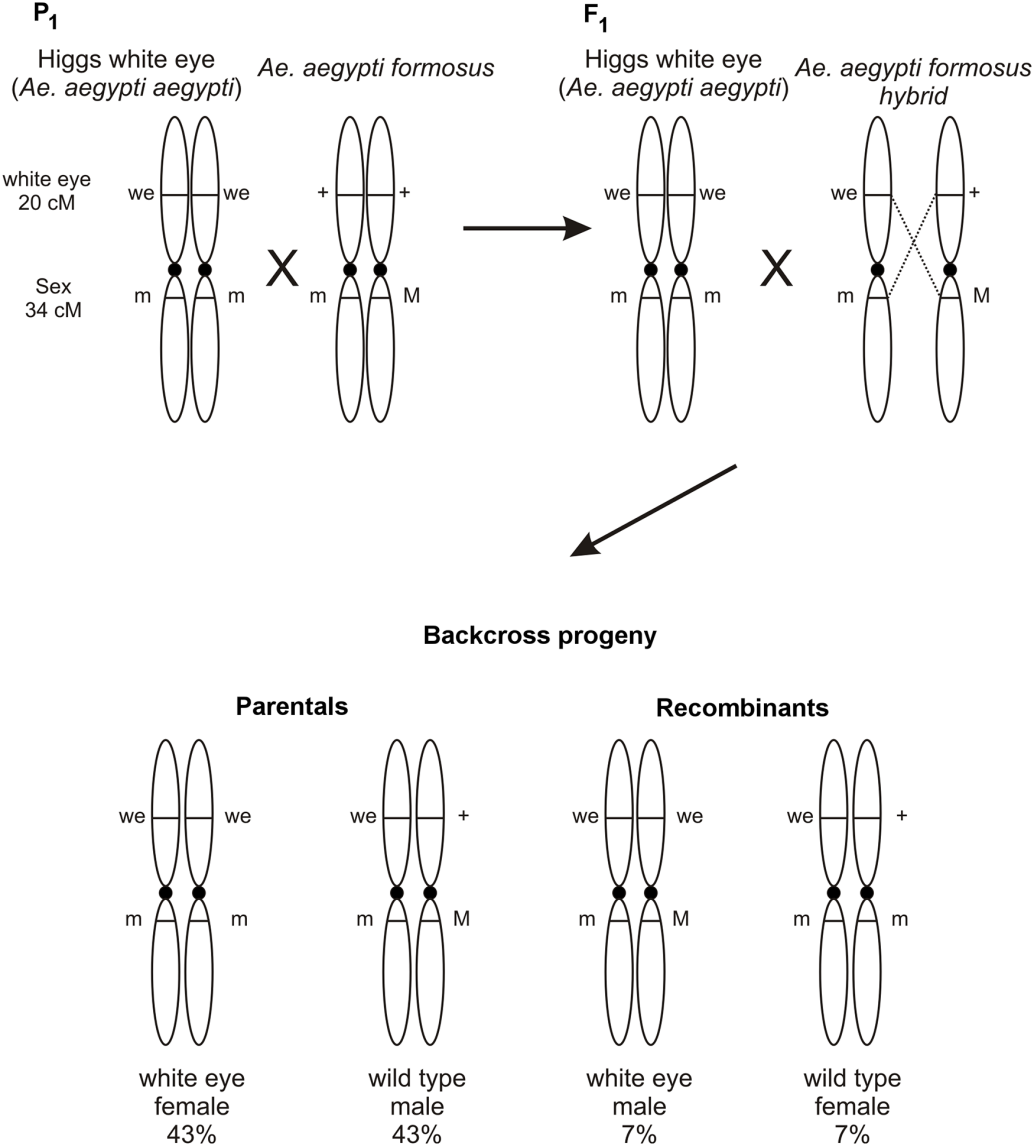
Crossing scheme used to assess the recombination rate between the *white-eye* (20 cM) and SDL (34 cM) markers on chromosome 1. The two loci were expected to have a recombination rate of 0.14. Male (M) alleles are dominant in *Aedes* so that males are M/m heterozygotes and females are m/m homozygous recessive. Wild type males +/+; M/m from a population of interest were crossed to a virgin HWE female (we/we; m/m) to produce F_1_ individuals heterozygous for the *white-eye* locus and the SDL. Male F_1_ were we/+; M/m which were backcrossed to virgin HWE females. Subsequent backcross progeny were scored for sex and eye color. Of the four possible phenotypes, *white-eye* females and wild-type males are parental genotypes, and white-eye males and wild type females are recombinant genotypes.

Prior studies document inversion polymorphisms on chromosome 1 in *Aaa* [54, 55]. The linkage distance of the white-eye marker [46] and the sex determining locus (SDL) were determined to test for chromosome rearrangements on chromosome 1. These markers are at 20 cM and 34 cM on the standard genetic map of chromosome 1 in *Ae*. *aegypti* [47]. This was done as a quick and inexpensive test for altered recombination rates, as would be expected if rearrangements exist on chromosome 1. Using the mating scheme outlined in Fig 11, 40 P_1_ families were set up in each of four West African collections and 20 families from a Mexican collection.

In each family the observed numbers of parental and recombinant backcross (BC) progeny were compared to expected numbers under one of three alternative genetic models (r = 14 cM; r = 0 cM (complete linkage); r ≥ 50 cM (unlinked)) using a χ^2^-goodness of fit test. Fig 1 show that r = 0.14 in 74% of 27 PK-10(F) families and r ≥ 0.5 in the remaining families. Amongst 27 Goudiry families r = 0.14 in 41% of families and r ≥ 0.5 in 59% of families. In Mont Rolland, in northwestern Senegal, r = 0.14 in 69% of 35 families, r ≥ 0.5 in 20% of families and r = 0.0 in 11% of families. Only 5 Bignona BC families survived and r = 0.0 in 4 of these and r ≥ 0.5 in one family. In the Vergel collection, 11 BC families produced > 20 offspring. Amongst these r = 0.14 in 9 families and r = 0.0 in two families. This study suggested widespread and abundant polymorphisms in chromosome 1 among *SenAae* populations however it does not suggest mechanisms to explain why r ≥ 0.5 between *white-eye* and SDL in many families and r = 0.0 in some families.

## Discussion

Over the last 15 years many F_1_ intercross families with various *Aedes* species have been constructed for use in QTL mapping experiments [56–65]. The same degree of sterility and low viability as seen in the present experiments was not observed in these earlier studies. An interesting and possibly unique aspect of this study concerns the ways that sterility became manifest as low oviposition rates in F_1_ and F_2_ females and poor egg-pupal-adult survival. Some of the fecundity results shown in Fig 2 are consistent with already well documented genetic differences in fecundity among *Ae*. *aegypti* strains [66]. That study also demonstrated that fecundity behaved as an additive quantitative genetic trait with hybrids (e.g. crosses d and e in Fig 2) having intermediate fecundities between the parents (e.g. crosses a and b in Fig 2). However the main reason for this drop in fecundity involved a failure to oviposit (Fig 2). Only one D2S3 mother failed to oviposit, while eight and nine females from PK10(G) x PK10(F) and the reciprocal cross failed to oviposit and five females failed to oviposit in both PK10(F) and PK10(G) families (Fig 2). The key observation is that PK10 females laid fewer eggs when crossed to a PK10 male but with a different abdominal scale pattern. Thus a large component of fecundity loss appears to be associated with a reduction in the tendency of *SenAae* females to oviposit under laboratory conditions and depended on the male with which they mated.

Haldane's rule [38] is one of the few empirical generalizations about speciation that holds true across different groups of animals and plants. Specifically, Haldane’s rule predicts that in fully or partially reproductively isolated species with heterogametic sex chromosomes, the heterogametic sex will be preferentially sterile and inviable in interspecific crosses. Dominance and rapid male evolution are often cited as the primary forces causing Haldane’s rule. Dominance is based upon the observation that alleles causing hybrid problems are most often deleterious and/or lethal recessives [67–70] and if they are sex-linked then heterogametic individuals, because they are hemizygous, will suffer their full effects. But deleterious and lethal recessives will be masked in the homogametic sex if one allele is wild-type. Amongst *Drosophila* species dominance appears to largely explain Haldane’s rule [71–73] for hybrid inviability. It has also been proposed that sexual selection may drive the more rapid evolution of male-expressed genes [74–76] as well as variation in expression of male-expressed genes [77]. More rapid male evolution would also explain Haldane’s rule because hybrid male sterility would occur before hybrid female sterility. Experiments in *Drosophila* suggest that faster male evolution causes Haldane’s rule for sterility [72, 78].

Presgraves and Orr [39] tested Haldane’s rule in mosquitoes, a family in which members of the subfamily *Anophelinae* have heterogametic sex chromosomes while species in the subfamily *Culicinae* (*Aedes*) have a sex determining locus (SDL) on an autosome. Male (“M”) alleles at the SDL are dominant so that males are “Mm” heterozygotes and females are homozygous recessive “mm.” Presgraves and Orr showed that for the most part Anopheline mosquitoes follow Haldane’s rule for sterility and inviability while *Aedes* mosquitoes only follow Haldane’s rule for sterility. The authors speculated that faster male evolution is driven by sexual selection which occurs irrespective of the sex determination mechanism. Alternatively, dominance is unlikely to act in *Aedes* because it lacks heterochromatic sex chromosomes and should exhibit little or no sex-limited inviability. Considering only reproductively isolated species and tabulating hybrid sterility and inviability separately, Presgraves and Orr classified the outcome of each interspecific cross as “male-affected”, “female affected”, or “both-sexes–affected.” *Anopheles* species obeyed Haldane’s rule for heterogametic sex while *Aedes* obeyed Haldane’s rule for hybrid sterility supporting the hypothesis that Haldane’s rule occurs even in species with autosomal sex determination. In all comparisons male-only hybrid sterility was noted, whereas female-only sterility was not seen. This also provided support for faster male evolution in *Aedes*. Sexual selection, which clearly operates in the genus *Aedes* [79, 80], seems a likely candidate that could drive more rapid male evolution.

However, most of the sterility in the present study arose from the absence of egg-laying, especially when females were crossed to hybrid F_1_ males. It was documented in 1958 that uninseminated *Ae*. *aegypti* females tend not to oviposit even when fully gravid [81]. In 1965, it was shown that male accessory gland substances, rather than sperm, acted as oviposition stimulants [82] in *Ae*. *aegypti*. Similarly in *Drosophila melanogaster*, the male accessory gland protein Acp26Aa stimulates oviposition [83–85]. It is possible that females in the present study failed to detect the male accessory gland proteins of a different species or subspecies and therefore failed to oviposit. This would be consistent with Presgraves and Orr suggestion that faster evolution occurs in male-specific genes. The *Ae*. *aegypti* semen proteome has been recently characterized [86–90] and reveals that while seminal fluid protein classes are conserved, many of the proteins evolve rapidly, necessitating identification of male reproductive gland proteins in each individual species.

Applying study designs similar to those used by McLain and Rai [79, 80] no evidence was found for prezygotic factors associated with the observed loss in fertility and survival. If the results of the insemination experiment with Bignona(G) and Bignona(F) (Fig 5) can be extended to the other *SenAae*, then there is no evidence for assortative mating or sperm inviability. But strong inferences cannot be made concerning assortative mating under laboratory conditions.

Chromosomal rearrangements have frequently been associated with speciation in taxa including mosquitoes [91]. Pericentric inversions are only rarely detected within animal species but can be abundant among species [92, 93]. Acentric fragments and dicentric “bridge” chromosomes arise when recombination occurs in parents that are heterozygous for pericentric inversions. These in turn yield aneuploid gametes and inviable zygotes. Therefore most F_1_ offspring arising from a cross between parents with (Goudiry Fig 9, Sedhiou Fig 10) and without pericentric (IB10) inversions are predicted to produce fewer viable larvae which will be homozygous for either of the two parental inversions.

The rearrangement on chromosome 2 was close to the centromere on the 2p arm. Positions of markers LF158 and a14 were found only in the standard arrangement in IB12 chromosomes (Fig 10A and 10B) and in the standard or standard/inverted arrangements (Fig 10C and 10D) in the Sedhiou and Goudiry strains. This result suggests that we found this inversion is only detectable in the heterozygote arrangement. Interestingly, the genetic marker LF158 is located at 36.7 cM, which is close to the position of the *Black-Tergite* genetic locus responsible for scaling patterns on the first abdominal tergite (34 cM) [47]. A major impediment to further cytogenetic studies was our inability to maintain *SenAae* isofemales lines. Although evidence of inversions and ribosomal gene duplications have been presented, maintenance of the strains with these rearrangements is difficult. Thus to date these rearrangements have not been validated nor have their geographic distributions been determined.

The large variation documented in recombination distances along chromosome 1 among *SenAae* collections (Fig 1) could occur through insertions, inversions or translocations. The variation could also occur because other genes elsewhere in the genome have come to control sex determination as has been noted for example in *Musca domestica* (L) populations [94]. Crosses between genotypes with different inversion types will have suppressed recombination rates, so if this was the cause then the original genetic map of *white-eye* could presumably have had an inversion polymorphism on chromosome 1 while crosses where r ≥ 0.5 would be the same inversion type. Further cytogenetic analyses and examination of additional collections will be needed to sort out these alternative explanations.

A critical question is whether all or some of our *SenAae* collections contain more than one species? Is hybrid sterility arising between two (or more) discrete subspecies? Alternatively are they occurring between individuals from the same collection? The critical test for cryptic species would be to identify *a priori* two or more types of mosquitoes based on genetic markers, cytogenetics or phenotype and then show that reproductive incompatibilities exist between these types and not within them. This has not been accomplished far for the many reasons listed above. Good candidates may be mtDNA clades [19, 25], chromosome rearrangements, independent linkage of *white-eye* and *SDL* (Fig 1) and still possibly scaling patterns on the first abdominal tergites (Figs 2 and 3).

An obvious question that remains is how *SenAae* are related to the *Aaf* and *Aaa* in East Africa. The earlier study of hybridization and mating behavior between *Aaa* and *Aaf* in East Africa concluded that these two forms are part of a single, albeit highly polytypic species [15]. The results of this study suggest that the taxa found in West Africa differ from the much more intensely studied *Aaa* and *Aaf* subspecies in East Africa. Assuming no pre-zygotic barriers and heterozygote breakdown (underdominance), collections that consist primarily of one taxon (few heterozygotes) are predicted to survive better than collections with more-or-less equal numbers of different taxa (many heterozygotes). Crosses within a cryptic species will produce offspring which survive to fully fertile adults. Even though individuals belonging to cryptic taxa can mate with one another, they produce few or no eggs (Fig 2) and if eggs are produced, they have lower survival to adults.

The suggestion that one or more new cryptic subspecies exist in Senegal in no way contradicts the phylogenetic patterns among worldwide *Ae*.*aegypti* s.l. populations that have been derived over the last 35 years using allozyme markers [20–22], microsatellites [16, 25, 26], nuclear SNP loci [15, 17, 27], mitochondrial DNA [19, 25] and most recently a SNP-Chip [17]. Instead our results suggest that taxa in some of the clades resolved in these phylogenetic studies may be partially or wholly reproductively isolated from one another and furthermore that some clades identified in these earlier studies may contain reproductively isolated taxa. The results presented here therefore serve as a reminder of the importance of evaluating reproductive isolation among closely related taxa rather than assuming that they belong to a single reproductively continuous population.

## Supporting Information

S1 Table Heterogeneity χ^2^ comparison of the numbers of females failing to oviposit in each of the ten crossing types. *P≤ 0.05, **P≤ 0.01, ***P≤ 0.0001.
(DOCX)

S2 Table Zero-inflated negative binomial (ZINB) regression to compare fecundity of all females in each of the ten crossing types. *P≤ 0.05, **P≤ 0.01, ***P≤ 0.0001.
(DOCX)

S3 Table Analysis of variance to compare fecundity of ovipositing females in each of the ten crossing types. The first line in each contrast are the degrees of freedom, the sum of squares, the mean square, F-values and the probability for comparison of the two crosses while the second line is the residual degrees of freedom, the residual sum of squares, and the residual mean square, *P≤ 0.05, **P≤ 0.01, ***P≤ 0.0001.
(DOCX)

S4 Table Analysis of variance to compare egg-pupal survival in each of the ten crossing types. The first line in each contrast are the degrees of freedom, the sum of squares, the mean square, F-values and the probability for comparison of the two crosses while the second line is the residual degrees of freedom, the residual sum of squares, and the residual mean square, *P≤ 0.05, **P≤ 0.01, ***P≤ 0.0001.
(DOCX)
