# Supplementary material for: Reproductive Incompatibility Involving Senegalese Aedes aegypti (L) Is Associated with Chromosome Rearrangements
Source: PLoS Negl Trop Dis. 2016 Apr 22;10(4):e0004626. doi: 10.1371/journal.pntd.0004626 (PMC4841568; doi:10.1371/journal.pntd.0004626)
Supplement: S1 Table — *P≤ 0.05, **P≤ 0.01, ***P≤ 0.0001. (DOCX) [file pntd.0004626.s001.docx]

S1 Table. Heterogeneity χ^2^ comparison of the numbers of females failing to oviposit in each of the ten crossing types. *P< 0.05, **P< 0.01, ***P< 0.0001

| Contrast | Cross | Proportion not ovipositing | Cross | Proportion not ovipositing | p-value |  |
| --- | --- | --- | --- | --- | --- | --- |
| Did ROCK females mated to Rock males oviposit more often than ROCK females mated to hybrid males? | | | | | |  |
| 1 | a) ROCK x ROCK | 6/40 | b) ROCK x (RxP) | 15/40 | 0.0406* | Yes |
| 2 | a) ROCK x ROCK | 6/40 | c) ROCK x (PxR) | 18/40 | 0.0066** | Yes |
| Did ROCK females mated to Rock males oviposit more often than hybrid females mated to ROCK males? | | | | | |  |
| 3 | a) ROCK x ROCK | 6/40 | d) (RxP) x ROCK | 14/40 | 0.0692 | No |
| 4 | a) ROCK x ROCK | 6/40 | e) (PxR) x ROCK | 2/40 | 0.2633 | No |
| Did ROCK females mated to hybrid males oviposit more often than hybrid females mated to ROCK males? | | | | | |  |
| 5 | b) ROCK x (RxP) | 15/40 | d) (RxP) x ROCK | 14/40 | 1 | No |
| 6 | c) ROCK x (PxR) | 18/40 | e) (PxR) x ROCK | 2/40 | 0.0001*** | Yes |
| Did PK10 females mated to PK10 males oviposit more often than PK10 females mated to hybrid males? | | | | | |  |
| 7 | f) PK10 x PK10 | 18/40 | g) PK10 x (RxP) | 23/40 | 0.3711 | No |
| 8 | f) PK10 x PK10 | 18/40 | h) PK10 x (PxR) | 12/40 | 0.2481 | No |
| Did PK10 females mated to PK10 males oviposit more often than hybrid females mated to PK10 males? | | | | | |  |
| 9 | f) PK10 x PK10 | 18/40 | i) (RxP) x PK10 | 3/40 | 0.0002*** | Yes |
| 10 | f) PK10 x PK10 | 18/40 | j) (PxR) x PK10 | 6/40 | 0.0066** | Yes |
| Did PK10 females mated to hybrid males oviposit more often than hybrid females mated to PK10 males? | | | | | |  |
| 11 | g) PK10 x (RxP) | 23/40 | i) (RxP) x PK10 | 3/40 | 0.0001*** | Yes |
| 12 | h) PK10 x (PxR) | 12/40 | j) (PxR) x PK10 | 6/40 | 0.1798 | No |
