# Supplementary material for: Reproductive Incompatibility Involving Senegalese Aedes aegypti (L) Is Associated with Chromosome Rearrangements
Source: PLoS Negl Trop Dis. 2016 Apr 22;10(4):e0004626. doi: 10.1371/journal.pntd.0004626 (PMC4841568; doi:10.1371/journal.pntd.0004626)
Supplement: S2 Table — *P≤ 0.05, **P≤ 0.01, ***P≤ 0.0001. (DOCX) [file pntd.0004626.s002.docx]

S2 Table. Zero-inflated negative binomial (ZINB) regression to compare fecundity of all females in each of the ten crossing types. *P< 0.05, **P< 0.01, ***P< 0.0001

| Contrast | Cross | Cross | Estimate | Std. Error | z value |  | Pr(>\|z\|) |  |
| --- | --- | --- | --- | --- | --- | --- | --- | --- |
| Did ROCK females mated to Rock males lay more eggs than ROCK females mated to hybrid males? | | | | | | | | |
|  | intercept |  | -1.7346 | 0.4428 | -3.917 |  | 0.0001*** | Yes |
| 1 | a) ROCK x ROCK | b) ROCK x (RxP) | 1.2238 | 0.5502 | 2.224 |  | 0.0261* | Yes |
| 2 | a) ROCK x ROCK | c) ROCK x (PxR) | 1.5339 | 0.5451 | 2.814 |  | 0.0049** | Yes |
| Did ROCK females mated to Rock males lay more eggs than hybrid females mated to ROCK males? | | | | | | | | |
| 3 | a) ROCK x ROCK | d) (RxP) x ROCK | 1.1156 | 0.5531 | 2.017 |  | 0.0437* | Yes |
| 4 | a) ROCK x ROCK | e) (PxR) x ROCK | -1.2098 | 0.8499 | -1.423 |  | 0.155 | No |
| Did ROCK females mated to hybrid males lay more eggs than hybrid females mated to ROCK males? | | | | | | | | |
|  | intercept |  | -0.5108 | 0.3266 | -1.564 |  | 0.118 | No |
| 5 | b) ROCK x (RxP) | d) (RxP) x ROCK | -0.1082 | 0.4654 | -0.233 |  | 0.114 | No |
|  | intercept |  | -0.2007 | 0.3178 | -0.631 |  | 0.5278 | No |
| 6 | c) ROCK x (PxR) | e) (PxR) x ROCK | -2.7438 | 0.792 | -3.464 |  | 0.0005*** | Yes |
| Did PK10 females mated to PK10 males lay more eggs than PK10 females mated to hybrid males? | | | | | | | | |
|  | intercept |  | -0.2007 | 0.3178 | -0.631 |  | 0.528 | No |
| 7 | f) PK10 x PK10 | g) PK10 x (RxP) | 0.4013 | 0.4495 | 0.893 |  | 0.372 | No |
| 8 | f) PK10 x PK10 | h) PK10 x (PxR) | -0.6466 | 0.4691 | -1.378 |  | 0.168 | No |
| Did PK10 females mated to PK10 males lay more eggs than hybrid females mated to PK10 males? | | | | | | | | |
| 9 | f) PK10 x PK10 | i) (RxP) x PK10 | -2.3116 | 0.6792 | -3.403 |  | 0.0007*** | Yes |
| 10 | f) PK10 x PK10 | j) (PxR) x PK10 | -1.5339 | 0.5451 | -2.814 |  | 0.0049** | Yes |
| Did PK10 females mated to hybrid males lay more eggs than hybrid females mated to PK10 males? | | | | | | | | |
|  | intercept |  | -0.2007 | 0.3178 | -0.631 |  | 0.5278 | No |
| 11 | g) PK10 x (RxP) | i) (RxP) x PK10 | -2.713 | 0.6792 | -3.994 |  | 0.0001*** | Yes |
|  | intercept |  | -0.8473 | 0.345 | -2.456 |  | 0.0141* | Yes |
| 12 | h) PK10 x (PxR) | j) (PxR) x PK10 | -0.8873 | 0.5614 | -1.581 |  | 0.114 | No |
