# Supplementary material for: Reproductive Incompatibility Involving Senegalese Aedes aegypti (L) Is Associated with Chromosome Rearrangements
Source: PLoS Negl Trop Dis. 2016 Apr 22;10(4):e0004626. doi: 10.1371/journal.pntd.0004626 (PMC4841568; doi:10.1371/journal.pntd.0004626)
Supplement: S3 Table — The first line in each contrast are the degrees of freedom, the sum of squares, the mean square, F-values and the probability for comparison of the two crosses while the second line is the residual degrees of freedom, the residual sum of squares, and the residual mean square, *P≤ 0.05, **P≤ 0.01, ***P≤ 0.0001. (DOCX) [file pntd.0004626.s003.docx]

S3 Table. Analysis of variance to compare fecundity of ovipositing females in each of the ten crossing types. The first line in each contrast are the degrees of freedom, the sum of squares, the mean square, F-values and the probability for comparison of the two crosses while the second line is the residual degrees of freedom, the residual sum of squares, and the residual mean square, *P< 0.05, **P< 0.01, ***P< 0.0001

| Contrast | Cross | Cross | d.f. | Sum Sq. | Mean Sq. | F value | Pr(>F) |  |
| --- | --- | --- | --- | --- | --- | --- | --- | --- |
| Did ROCK females mated to Rock males lay more eggs than ROCK females mated to hybrid males? | | | | | | | | |
| 1 | a) ROCK x ROCK | b) ROCK x (RxP) | 1 | 3.59 | 3.59 | 15.75 | 0.0002*** | Yes |
|  |  |  | 57 | 13 | 0.23 |  |  |  |
| 2 | a) ROCK x ROCK | c) ROCK x (PxR) | 1 | 0.27 | 0.27 | 5.28 | 0.0254* | Yes |
|  |  |  | 55 | 2.81 | 0.05 |  |  |  |
| Did ROCK females mated to Rock males lay more eggs than hybrid females mated to ROCK males? | | | | | | | | |
| 3 | a) ROCK x ROCK | d) (RxP) x ROCK | 1 | 5.62 | 5.62 | 35.61 | 0.0001*** | Yes |
|  |  |  | 58 | 9.16 | 0.16 |  |  |  |
| 4 | a) ROCK x ROCK | e) (PxR) x ROCK | 1 | 5.7 | 5.7 | 36.44 | 0.0001*** | Yes |
|  |  |  | 70 | 10.94 | 0.16 |  |  |  |
| Did ROCK females mated to hybrid males lay more eggs than hybrid females mated to ROCK males? | | | | | | | | |
| 5 | b) ROCK x (RxP) | d) (RxP) x ROCK | 1 | 0.18 | 0.18 | 0.52 | 0.474 | No |
|  |  |  | 49 | 16.86 | 0.34 |  |  |  |
| 6 | c) ROCK x (PxR) | e) (PxR) x ROCK | 1 | 0.67 | 0.67 | 2.07 | 0.155 | No |
|  |  |  | 58 | 18.75 | 0.32 |  |  |  |
| Did PK10 females mated to PK10 males lay more eggs than PK10 females mated to hybrid males? | | | | | | | | |
| 7 | f) PK10 x PK10 | g) PK10 x (RxP) | 1 | 0.05 | 0.05 | 0.12 | 0.733 | No |
|  |  |  | 38 | 14.95 | 0.39 |  |  |  |
| 8 | f) PK10 x PK10 | h) PK10 x (PxR) | 1 | 0.37 | 0.37 | 1.26 | 0.267 | No |
|  |  |  | 48 | 14.13 | 0.29 |  |  |  |
| Did PK10 females mated to PK10 males lay more eggs than hybrid females mated to PK10 males? | | | | | | | | |
| 9 | f) PK10 x PK10 | i) (RxP) x PK10 | 1 | 1.76 | 1.75 | 5.94 | 0.0180* | Yes |
|  |  |  | 57 | 16.85 | 0.3 |  |  |  |
| 10 | f) PK10 x PK10 | j) (PxR) x PK10 | 1 | 0.17 | 0.17 | 0.51 | 0.48 | No |
|  |  |  | 54 | 18.63 | 0.34 |  |  |  |
| Did PK10 females mated to hybrid males lay more eggs than hybrid females mated to PK10 males? | | | | | | | | |
| 11 | g) PK10 x (RxP) | i) (RxP) x PK10 | 1 | 1.01 | 1.01 | 5.98 | 0.0178* | Yes |
|  |  |  | 53 | 8.92 | 0.17 |  |  |  |
| 12 | h) PK10 x (PxR) | j) (PxR) x PK10 | 1 | 0.05 | 0.05 | 0.33 | 0.569 | No |
|  |  |  | 60 | 9.88 | 0.16 |  |  |  |
