# Supplementary material for: Reproductive Incompatibility Involving Senegalese Aedes aegypti (L) Is Associated with Chromosome Rearrangements
Source: PLoS Negl Trop Dis. 2016 Apr 22;10(4):e0004626. doi: 10.1371/journal.pntd.0004626 (PMC4841568; doi:10.1371/journal.pntd.0004626)
Supplement: S4 Table — The first line in each contrast are the degrees of freedom, the sum of squares, the mean square, F-values and the probability for comparison of the two crosses while the second line is the residual degrees of freedom, the residual sum of squares, and the residual mean square, *P≤ 0.05, **P≤ 0.01, ***P≤ 0.0001. (DOCX) [file pntd.0004626.s004.docx]

S4 Table. Analysis of variance to compare egg-pupal survival in each of the ten crossing types. The first line in each contrast are the degrees of freedom, the sum of squares, the mean square, F-values and the probability for comparison of the two crosses while the second line is the residual degrees of freedom, the residual sum of squares, and the residual mean square, *P< 0.05, **P< 0.01, ***P< 0.0001

| Contrast | Cross | Cross | d.f. | Sum Sq. | Mean Sq. | F value | Pr(>F) |  |
| --- | --- | --- | --- | --- | --- | --- | --- | --- |
| Did the offspring of ROCK x ROCK crosses have better survival than offspring of ROCK x hybrid crosses? | | | | | | | | |
| 1 | a) ROCK x ROCK | b) ROCK x (RxP) | 1 | 0.39 | 0.39 | 6.99 | 0.0105* | Yes |
|  |  |  | 58 | 3.27 | 0.06 |  |  |  |
| 2 | a) ROCK x ROCK | c) ROCK x (PxR) | 1 | 8.18 | 8.18 | 33.73 | 0.0001*** | Yes |
|  |  |  | 54 | 13.1 | 0.24 |  |  |  |
| Did the offspring of ROCK x ROCK crosses have better survival than offspring of hybrid x ROCK crosses? | | | | | | | | |
| 3 | a) ROCK x ROCK | d) (RxP) x ROCK | 1 | 0.21 | 0.21 | 4.57 | 0.0368* | Yes |
|  |  |  | 59 | 2.67 | 0.05 |  |  |  |
| 4 | a) ROCK x ROCK | e) (PxR) x ROCK | 1 | 0.71 | 0.71 | 16.05 | 0.0002*** | Yes |
|  |  |  | 71 | 3.13 | 0.04 |  |  |  |
| Did the offspring of ROCK x hybrid crosses have better survival than offspring of hybrid x ROCK crosses? | | | | | | | | |
| 5 | b) ROCK x (RxP) | d) (RxP) x ROCK | 1 | 1.02 | 1.01 | 14.34 | 0.0004*** | Yes |
|  |  |  | 49 | 3.47 | 0.07 |  |  |  |
| 6 | c) ROCK x (PxR) | e) (PxR) x ROCK | 1 | 1.59 | 1.59 | 26.7 | 0.0001*** | Yes |
|  |  |  | 58 | 3.46 | 0.06 |  |  |  |
| Did the offspring of PK10 x PK10 crosses have better survival than offspring of PK10 x hybrid crosses? | | | | | | | | |
| 7 | f) PK10 x PK10 | g) PK10 x (RxP) | 1 | 0.05 | 0.05 | 0.71 | 0.403 | No |
|  |  |  | 38 | 2.63 | 0.07 |  |  |  |
| 8 | f) PK10 x PK10 | h) PK10 x (PxR) | 1 | 0.02 | 0.02 | 0.28 | 0.602 | No |
|  |  |  | 48 | 4.06 | 0.08 |  |  |  |
| Did the offspring of PK10 x PK10 crosses have better survival than offspring of hybrid x PK10 crosses? | | | | | | | | |
| 9 | f) PK10 x PK10 | i) (RxP) x PK10 | 1 | 0.3 | 0.3 | 1.81 | 0.184 | No |
|  |  |  | 57 | 9.37 | 0.16 |  |  |  |
| 10 | f) PK10 x PK10 | j) (PxR) x PK10 | 1 | 0.01 | 0.01 | 0.17 | 0.683 | No |
|  |  |  | 54 | 3.93 | 0.07 |  |  |  |
| Did the offspring of PK10 x hybrid crosses have better survival than offspring of hybrid x PK10 crosses? | | | | | | | | |
| 11 | g) PK10 x (RxP) | i) (RxP) x PK10 | 1 | 0.07 | 0.07 | 0.49 | 0.486 | No |
|  |  |  | 53 | 7.58 | 0.14 |  |  |  |
| 12 | h) PK10 x (PxR) | j) (PxR) x PK10 | 1 | 0.08 | 0.08 | 1.41 | 0.241 | No |
|  |  |  | 60 | 3.57 | 0.06 |  |  |  |
